# Species-Level Variability in Extracellular Production Rates of Reactive Oxygen Species by Diatoms

**DOI:** 10.3389/fchem.2016.00005

**Published:** 2016-03-30

**Authors:** Robin J. Schneider, Kelly L. Roe, Colleen M. Hansel, Bettina M. Voelker

**Affiliations:** ^1^Department of Chemistry, St. John's UniversityNew York, NY, USA; ^2^Department of Chemistry and Geochemistry, Colorado School of MinesGolden, CO, USA; ^3^Department of Marine Chemistry and Geochemistry, Woods Hole Oceanographic InstitutionWoods Hole, MA, USA

**Keywords:** reactive oxygen species, superoxide, hydrogen peroxide, diatoms, culture

## Abstract

Biological production and decay of the reactive oxygen species (ROS) hydrogen peroxide (H_2_O_2_) and superoxide (O${}_{2}^{-}$) likely have significant effects on the cycling of trace metals and carbon in marine systems. In this study, extracellular production rates of H_2_O_2_ and O${}_{2}^{-}$ were determined for five species of marine diatoms in the presence and absence of light. Production of both ROS was measured in parallel by suspending cells on filters and measuring the ROS downstream using chemiluminescence probes. In addition, the ability of these organisms to break down O${}_{2}^{-}$ and H_2_O_2_ was examined by measuring recovery of O${}_{2}^{-}$ and H_2_O_2_ added to the influent medium. O${}_{2}^{-}$ production rates ranged from undetectable to 7.3 × 10^−16^ mol cell^−1^ h^−1^, while H_2_O_2_ production rates ranged from undetectable to 3.4 × 10^−16^ mol cell^−1^ h^−1^. Results suggest that extracellular ROS production occurs through a variety of pathways even amongst organisms of the same genus. *Thalassiosira* spp. produced more O${}_{2}^{-}$ in light than dark, even when the organisms were killed, indicating that O${}_{2}^{-}$ is produced via a passive photochemical process on the cell surface. The ratio of H_2_O_2_ to O${}_{2}^{-}$ production rates was consistent with production of H_2_O_2_ solely through dismutation of O${}_{2}^{-}$ for *T. oceanica*, while *T. pseudonana* made much more H_2_O_2_ than O${}_{2}^{-}$. *T. weissflogii* only produced H_2_O_2_ when stressed or killed. *P. tricornutum* cells did not make cell-associated ROS, but did secrete H_2_O_2_-producing substances into the growth medium. In all organisms, recovery rates for killed cultures (94–100% H_2_O_2_; 10–80% O${}_{2}^{-}$) were consistently higher than those for live cultures (65–95% H_2_O_2_; 10–50% O${}_{2}^{-}$). While recovery rates for killed cultures in H_2_O_2_ indicate that nearly all H_2_O_2_ was degraded by active cell processes, O${}_{2}^{-}$ decay appeared to occur via a combination of active and passive processes. Overall, this study shows that the rates and pathways for ROS production and decay vary greatly among diatom species, even between those that are closely related, and as a function of light conditions.

## Introduction

The reactive oxygen species (ROS), superoxide radical (O${}_{2}^{-}$), hydrogen peroxide (H_2_O_2_), and hydroxyl radical (OH) are intermediates in the sequential one-electron reduction of oxygen to water, and are important to the biogeochemical cycling of trace metals and carbon.

Photochemical production of O${}_{2}^{-}$ in the marine environment has been well-studied, and occurs when photo-excited chromophoric dissolved organic matter (CDOM) transfers an electron to dissolved O_2_ to generate O${}_{2}^{-}$ (Cooper et al., 1988; Shaked et al., 2010). Biological production of O${}_{2}^{-}$ also occurs in marine environments, but is less well-understood than photochemical production (Rose et al., 2010). The typical removal pathways for O${}_{2}^{-}$ are by a dismutation reaction (Cooper and Zika, 1983; Zafiriou, 1990) and by redox reactions with trace metals and organic matter (Goldstone and Voelker, 2000; Wuttig et al., 2013).

H_2_O_2_ is produced through dismutation and reduction of O${}_{2}^{-}$; it therefore has the same photochemical and biological sources as O${}_{2}^{-}$ (Zhang et al., 2012). In addition, H_2_O_2_ can be produced biologically without O${}_{2}^{-}$ as a precursor (Palenik et al., 1987). H_2_O_2_ can decompose through reaction with reduced metals to form OH; however, in marine environments, the predominant method of decay is likely to be enzymatic destruction (Petasne and Zika, 1997; Herut et al., 1998; Yuan and Shiller, 2001).

Field studies have shown that particle-associated production of ROS occurs in the ocean (Avery et al., 2005; Rose et al., 2008; Hansard et al., 2010) and that this production can be slowed by biological inhibitors (Moffett and Zafiriou, 1990; Rose et al., 2010), indicating that it is of biological origin. Recent studies by Vermilyea et al. (2010) and Roe et al. (2016) show that dark production of H_2_O_2_ in the Gulf of Alaska and at Station ALOHA is significant compared to photochemical production, indicating that biological ROS production may impact biogeochemical cycles in the ocean. Thus, it is important to consider which organisms produce ROS, how they do so, and why.

Most culture studies of biological extracellular ROS production have been performed on ichthyotoxic organisms that negatively impact the fishing industry. *Chattonella marina*, in particular, along with other raphidophyte species, have been studied intensively (see list in Marshall et al., 2002). However, from a global perspective, it is interesting to consider the more common phytoplankton—diatoms, coccolithophores, and cyanobacteria—which are more likely to influence biological production of ROS in the majority of the ocean. Extracellular production rates have been quantified in only a few species to date (see Table 1 and references cited therein): the diatoms *Thalassiosira pseudonana* and *T. weissflogii*, coccolithophore *Pleurochrysis carterae*, and cyanobacteria *Synechococcus* sp., *Lyngbya majuscula* and *Anacystis nidulans*. Compared to raphidophytes, these species of phytoplankton have cell normalized O${}_{2}^{-}$ and H_2_O_2_ production rates that are up to five orders of magnitude lower (Table 1).

**Table 1:** **Previously published phytoplankton studies showing cell-normalized production of superoxide (P_***O***2−, ***cell***_) and hydrogen peroxide (P_***H***22, ***cell***_)**.

**Organism**	**Type**	**Study**	**P_*O*2−, *cell*_(10^−17^ mol cell^−1^ h^−1^)**	**P_*H*2*O*2, *cell*_(10^−17^ mol cell^−1^ h^−1^)**
*A. nidulans*	Cyanobacterium	Scholz et al., 1995	–	500
*Synechococcus*	Cyanobacterium	Rose et al., 2008	4–10	N/A
*H. carterae*	Coccolithophore	Palenik et al., 1987	ND	1–2 × 10^3^
*T. weissflogii*	diatom	Palenik et al., 1987	–	ND
		Milne et al., 2009	25–132	11–66
		Kustka et al., 2005	84	–
		Rose et al., 2008	80–140	–
*T. pseudonana*	Diatom	Rose et al., 2008	40–83	–
		Waring et al., 2010	–	7–14
*C. antiqua*	Raphidophyte	Oda et al., 1997	6.6 × 10^5^	8.0 × 10^4^
*C. marina*		Oda et al., 1997	1.6 × 10^6^	5.2 × 10^5^
		Yamasaki et al., 2004	–	2.8 × 10^6^
*K. mikimotoi*		Yamasaki et al., 2004	–	2.6 × 10^5^
*H. akashiwo*		Twiner and Trick, 2000	–	1.8 × 10^3^
*O. luteus*		Kim et al., 1999	6.6 × 10^3^	1.0 × 10^4^
*Symbiodinium* sp.	Algal symbiont	Saragosti et al., 2010	288–372	–
*S. goreaui*		Saragosti et al., 2010	1.7–7.4 × 10^3^	–

Dashes indicate that the study did not examine production of that ROS; ND indicates that the researchers attempted to measure the ROS but did not detect it. Although Lyngbya majuscula was found to produce O_2-_ (Rose et al., 2005), no cell-normalized production numbers are available, so it is not included.

Previous studies suggest that ROS production may occur for different reasons in different organisms. Palenik and Morel (1990) showed that *H. carterae* produced H_2_O_2_ as a byproduct of uptake of organic nitrogen sources. Two organisms, *Trichodesmium* (Roe and Barbeau, 2014) and *Lyngbya majuscula* (Rose et al., 2005) have been postulated to use O${}_{2}^{-}$ as a reductant to facilitate biological uptake of iron. In contrast, production of superoxide was not beneficial for iron uptake by *T. weissflogii* (Kustka et al., 2005); an alternative explanation for superoxide production by *T. weissflogii* has not been proposed. Alternatively, O${}_{2}^{-}$ has also been proposed as a cell signal and autocrine growth promoter in *C. marina* and *Prymnesium parum* that is required for cell proliferation (Oda et al., 1995; Marshall et al., 2005).

Extracellular H_2_O_2_ could be produced simply via dismutation or reduction of biologically produced O${}_{2}^{-}$, or due to direct production by a separate mechanism. Thus, one way to gain a better understanding of H_2_O_2_ production mechanisms is to determine the relationship between extracellular O${}_{2}^{-}$ and H_2_O_2_ production rates. Of the previous studies on ROS production by non-raphidophytes, only two (Palenik et al., 1987; Milne et al., 2009) measured both species directly. In the first study, *P. carterae* produced H_2_O_2_ without measurable O${}_{2}^{-}$ during uptake of organic nitrogen (Palenik et al., 1987). By contrast, the ratio between O${}_{2}^{-}$ and H_2_O_2_ production by *T. weissflogii* under high light conditions was around the 2:1 ratio expected for production of H_2_O_2_ via the superoxide dismutation pathway (Milne et al., 2009).

Direct comparisons of rates of biological production of both O${}_{2}^{-}$ and H_2_O_2_ under light and dark conditions are important for better understanding factors that stimulate production, determining links between production and photosynthesis, and identifying possible sources of ROS to the dark ocean. Milne et al. (2009) showed increased extracellular production of O${}_{2}^{-}$ by *T. weissflogii* under high light conditions (150–500 μmol photons m^−2^ s^−1^), and Waring et al. (2010) showed increased H_2_O_2_ production by *T. pseudonana* under high light (1000 μmol photons m^−2^ s^−1^). Nevertheless, enhanced O${}_{2}^{-}$ production cannot be a direct byproduct of photosynthesis, as O${}_{2}^{-}$ cannot pass the cell membrane (Seaver and Imlay, 2001). Instead, stimulated photosynthetic activity would lead to increased intracellular NADPH pools that could serve as a source of reducing equivalents to transmembrane NADPH oxidoreductases proposed to be involved in extracellular superoxide production (see for instance, Kawano et al., 1996). Another possibility that has not been examined is that light-induced ROS production is due to a passive biological mechanism (e.g., outer membrane proteins and/or pigments) on the cell surface; if so, ROS production in the light could continue even after cell death.

Finally, ROS production rates are typically reported as net production rates, which do not take into account the contribution of decay to ROS concentrations. Diaz et al. (2013) showed that net O${}_{2}^{-}$ production rates by heterotrophic bacteria could be significantly affected by decay. Thus, to obtain gross production rates, decay, and production rates should be addressed in conjunction with each other. In addition, although Wong et al. (2003) found that killed organisms decompose substantially less H_2_O_2_ than live ones, no study has yet determined whether there is a similar effect on O${}_{2}^{-}$ decomposition.

The goals of this study were to measure O${}_{2}^{-}$ and H_2_O_2_ production by common phytoplankton, to examine whether there is a link between the two ROS, and to determine the influence of light and active metabolism on their production and decay rates. We focused on five species of diatoms, three of which were in the same genus. Production of both ROS was measured in parallel by both live and killed cultures under light and dark conditions. In addition to measuring production rates, we also determined recoveries of the ROS during measurement. This not only allowed us to quantify gross, rather than net, production, but also served as a measurement of the relative ability of these organisms to break down ROS under different conditions.

## Materials and methods

### Organisms: growth and experimental conditions

Five marine diatom species were used in this study: *Thalassiosira pseudonana* (CCMP 1335), *Phaeodactylum tricornutum, Cyclotella cryptica* (CCMP 332), *Thalassiosira oceanica* (CCMP 1005), and *Thalassiosira weissflogii* (CCMP 1336). Axenic cultures were obtained from the National Center for Marine Algae (NCMA) and from the Hildebrand lab at Scripps Institute of Oceanography. Maintenance cultures were grown in acid-washed polycarbonate flasks in Guillard's F/2 medium (Sigma) under grow lights at 20°C at 100 μmol photons m^−2^s^−1^ (as measured by a LI-COR LI-250 light meter and LI-190 Quantum/PAR sensor) with a 12 h light:dark cycle. Maintenance cultures were transferred to fresh medium every 2 weeks. An aliquot was taken from the maintenance culture during exponential growth to start the experimental culture, which was grown to mid-exponential phase under the same conditions in F/2 medium before being harvested for the experiment. On the day of the experiment, an aliquot of experimental culture was removed for measuring cell density on a Beckman Z2 Coulter counter, and all cultures were checked for purity with marine purity broth (Saito et al., 2002) or, for *P. tricornutum*, by making slides of DAPI-stained cultures.

Cells were harvested for each experimental run by removing an aliquot (~5 mL to get ~10^6^ cells) of the experimental culture from the light and loading it directly onto an acid-washed (0.1 M HCl) 25-mm 0.45-μm cellulose acetate syringe filter (VWR) as described below. The total number of cells on each filter was estimated based on multiplying the cell density per mL by the volume of culture loaded. The filter disk was positioned parallel to the floor for the whole experiment. For H_2_O_2_ measurements, cells were generally loaded onto the filter using a peristaltic pump (0.6 mL min^−1^). In a few experiments, cells were gently loaded using a syringe (~5 mL/min). For O${}_{2}^{-}$ measurements, the culture was loaded onto the filter using a peristaltic pump at 3 mL min^−1^, and the pump was briefly stopped (<2 s) while the tubing was moved from the ASW bottle to the culture bottle to avoid trapping air on the filter.

For both O${}_{2}^{-}$ and H_2_O_2_ measurements, the entire experimental run was either conducted under light conditions (from a small grow light emitting 75 μmol photons m^−2^ s^−1^ as measured by a LI-COR LI-250 light meter and LI-190 Quantum/PAR sensor held directly adjacent to the filter) or in the dark (wrapped in foil). The analytical medium was an artificial seawater (hereafter referred to as ASW) based on a modified Aquil medium (Price et al., 1989) where only the major salts were added and chelexed, adjusted to pH 8, and amended with 10 μM of the chelator diethylenetriaminepentaacetic acid (DTPA) (Sigma), which was added to prevent rapid loss of O${}_{2}^{-}$ via reactions with trace metals.

A 4% formaldehyde killed control was allowed to incubate for ~2 h before the cells were rinsed on a 2.0 μm filter and resuspended in fresh F/2 medium. The killed culture was then placed back in the growing conditions for ~1 h before being harvested for the experiment. A second killed control was maintained in the growth conditions for a week to ensure that this procedure successfully killed each organism.

Each organism was analyzed on two separate days, and on each day, six experimental runs were performed: two runs with live organisms kept in the dark; two runs with live organisms in the light; and one run each with killed organisms in the light and in the dark.

### O${}_{2}^{-}$ detection

O${}_{2}^{-}$ was detected with a flow injection system (FeLume Mini, Waterville Analytical) with the use of the MCLA chemiluminescence probe (Godrant et al., 2009) using the experimental set-up described in Section S1.1 (Figure S1).

Calibration of the instrument was completed at the start of each experimental day using ASW. An acid-washed 25-mm 0.45-μm cellulose acetate filter was used during calibration to mimic runs in the presence of cells as closely as possible. Briefly, primary O${}_{2}^{-}$ stock solutions were prepared with KO_2_ and calibrated spectrophotometrically. These were then diluted to make working stock solutions, which were added to the ASW (see Section S1.2 for details). The output signal was then monitored as a function of time and fit to:

(1) $$R_{t}\text{​}=\text{​}R_{BL}\;+\;R_{t=0}exp^{\left(-k_{loss,O2-}t\right)}$$

where *R*_*t*_ is the measured response at time *t, R*_*BL*_ is the baseline response, *R*_*t* = 0_ is the baseline corrected response at *t* = 0, and *k*_*loss, O*2−_ is the pseudo-first order decay constant in the analytical medium. The sensitivity (*S*, counts nM^−1^) of the response was determined as:

(2) $$S\;=\;\frac{R_{t\;=\;0}}{{\left[O_{2}^{-}\right]}_{spike}}$$

An average of four S measurements was used for each calibration. [*O*${}_{2}^{-}$]_*spike*_ generally ranged from 0.207 to 1.079 nM. No dependence of S on [*O*${}_{2}^{-}$]_*spike*_ was observed and all of the decay curves were well-described by first-order kinetics.

### Calculation of O${}_{2}^{-}$ production and percent recovery from cultures

An experimental run consisted of the following steps: measuring the steady-state signal R_*ASW*_ from running ASW past an empty filter, loading cells on the filter, measuring the steady-state signal *R*_*cell*_ from running ASW past the cells on the filter, observing the signal response to a known O${}_{2}^{-}$ spike to determine the recovery of O${}_{2}^{-}$ in the presence of cells, and finally measuring the steady-state background signal from MCLA autooxidation, R_*SOD*_, after addition of ~0.24 U mL ^−1^ (~1.6 nM) SOD to the ASW (Section S1.3, Figure S2).

Recovery *Rec*_*O*2−_ was determined by fitting the spike data to Equation (1), with *R*_*BL*_fixed to the measured value of *R*_*cell*_. The recovery (*Rec*_*O*2−_) was then calculated from:

(3) $$Rec_{O2-}\;=\;\left(\frac{R_{t=0}}{Expected\;R_{t=0}}\right)$$

where Expected *R*_*t* = 0_ was calculated as the product of sensitivity S and the concentration of the added O${}_{2}^{-}$ spike.

The increase in O${}_{2}^{-}$ concentration due to production by the cells, [*O*${}_{2}^{-}$]_*cell*_ (nM), was calculated from:

(4) $${\left[O_{2}^{-}\right]}_{cell}=\left(\frac{R_{cell}-R_{SOD}}{\text{S }\times \text{ RecO2}-}\right)-\;{\left[O_{2}^{-}\right]}_{ASW}$$

where the first term represents the O${}_{2}^{-}$ concentration measured in the presence of cells and the second term, [*O*${}_{2}^{-}$]_*ASW*_, is the background concentration of O${}_{2}^{-}$ in the ASW. [*O*${}_{2}^{-}$]_*ASW*_ was calculated according to:

(5) $${\left[O_{2}^{-}\right]}_{ASW}=(\frac{{\text{R}}_{\text{ASW}}-{\text{R}}_{\text{SOD}}}{S})$$

The cell-normalized production rate *P*_*O*2−, *cell*_ (nmol cell^−1^ h^−1^) was determined by:

(6) $$P_{O2-}=\;\frac{{\left[O_{2}^{-}\right]}_{cell}\times \;Q}{N}\;\times \;(3600\;s\;hr^{-1})$$

where *Q* is the flow rate (L s^−1^) and N is the total number of cells on the filter.

Our calculations of [*O*${}_{2}^{-}$]_*cell*_ and [*O*${}_{2}^{-}$]_*ASW*_ above assume that the difference between R_*ASW*_ and R_*SOD*_ is due only to a background concentration of O${}_{2}^{-}$ in the ASW. We have tested this assumption in another work (Roe, unpublished data), where we observed that small (<0.1 nM) additions of SOD to the ASW bottle decreased R_*ASW*_ proportionally to the resulting increase in O${}_{2}^{-}$ decay rate. This gradual decrease in R_*ASW*_-R_*SOD*_ with increasing SOD concentration can therefore be attributed to a “bottle blank” created by a constant background production rate of O${}_{2}^{-}$ in the ASW bottle, which could be due to compounds entering from the atmosphere or to redox reactions occurring at the bottle wall or in the ASW. The final large addition of SOD to the ASW bottle to establish R_*SOD*_ caused an additional small decrease in signal, indicating that there is another effect of SOD beyond eliminating the “bottle blank” O${}_{2}^{-}$, which we will call a method blank. By not accounting for the method blank separately, we are essentially assuming that it will be affected by the decreased recovery in the presence of cells the same way that the O${}_{2}^{-}$ bottle blank is affected. However, because the method blank is small, this assumption does not add much uncertainty to our reported measurements. The method blank in DTPA-amended ASW corresponded to a contribution to [*O*${}_{2}^{-}$]_*ASW*_ (as defined by Equation 5) of 0.015 ± 0.006 nM (*n* = 7) (Roe, unpublished data). If the method blank in the present study is similar to that measured by Roe et al., but not affected by recovery, our reported values of [*O*${}_{2}^{-}$]_*cell*_ would represent an overestimate of 6 ± 4%.

We define the detection limit for each run as five times the standard deviation of the baseline signal. Detection limits converted to concentration by dividing by S ranged from 0.013 to 0.092 nM. In several samples, the signal from the spike did not give a detectable offset from *R*_*cell*_. In these cases, *Rec*_*O*2−_, [*O*${}_{2}^{-}$]_*cell*_, and *P*_*O*2−_ could not be determined.

### H_2_O_2_ detection and calibration

H_2_O_2_ was measured by flow injection analysis using a Waterville Analytical FeLume system and the base-catalyzed chemiluminescent reaction with acridinium ester as described by Cooper et al. (2000) and King et al. (2007). Briefly, a slug (~0.5 mL) of sample was pushed into the system by a stream consisting of an artificial seawater (ASW) (Price et al., 1989) treated with 10 U/mL catalase. The sample then combined with the acridinium ester reagent (10 μM, pH 3) in a mixing tee. Next the sample/acridinium ester mixture entered a flow cell, where it mixed with carbonate buffer (0.02 M, pH 11.2). The photons produced by the reaction were measured by a photomultiplier tube (Section S2, Figure S3).

A calibration curve was created at the beginning of each experimental day by standard additions of H_2_O_2_ stock to aliquots of ASW. H_2_O_2_ stock solutions were prepared immediately before calibration by dilution of a ~3 mM primary stock solution that was stored in the refrigerator. The concentration of the primary stock solution was determined approximately every 2 months by measuring its absorbance at 240 nM, at which the molar absorptivity of H_2_O_2_ is 38.1 ± 1.4 M^−1^ cm^−1^ (Miller and Kester, 1988). The loss of H_2_O_2_ in the primary stock solution over the course of the study was less than 1%.

### Calculation of production rates and percent recovery for H_2_O_2_

For each experimental run, filter-sterilized ASW flowed through a peristaltic pump at 0.6 mL min^−1^ over an empty acid-washed (0.1 M HCl) filter and directly into the FeLume system until a steady-state concentration of H_2_O_2_, representing a background concentration, was detected ([*H*_2_*O*_2_]_*unspiked, direct*_). Next, the cells were loaded onto the filter as described above, and the H_2_O_2_ was again monitored until a steady state was reached ([*H*_2_*O*_2_]_*unspiked, cells*_). Then the ASW was spiked with additional H_2_O_2_ and flowed over the cells and measured ([*H*_2_*O*_2_]_*spiked, direct*_). Finally, the filter was disconnected from the ASW stream and the H_2_O_2_ of the spiked ASW was quantified ([*H*_2_*O*_2_]_*spiked, direct*_). An example of a typical experimental run is shown in Figure S4.

Recovery (*Rec*_*H*2*O*2_) for each experimental run was calculated by dividing the increase in [H_2_O_2_] in the medium due to the addition of the H_2_O_2_ spike by the measured increase in the presence of cells:

(7) $$Rec_{H2O2}=\frac{{\left[H_{2}O_{2}\right]}_{spiked,\;cells}-{\left[H_{2}O_{2}\right]}_{unspiked,\;cells}}{{\left[H_{2}O_{2}\right]}_{spiked,\;direct}-{\left[H_{2}O_{2}\right]}_{unspiked,\;direct}}$$

Calculation of recovery in this manner assumes that the cells on the filter break down the same fraction of H_2_O_2_ from both the spiked and unspiked medium, i.e., that the decomposition is first order in H_2_O_2_.

The increase in [H_2_O_2_] due to cell production, [*H*_2_*O*_2_]_*cell*_ (nmol L^−1^) for a given experimental run was then calculated with the equation:

(8) $${\left[H_{2}O_{2}\right]}_{cell}=\frac{{\left[H_{2}O_{2}\right]}_{unspiked,\;cells}}{Rec_{H2O2}}\;-{\left[H_{2}O_{2}\right]}_{unspiked,\;direct}$$

A cell-normalized production rate *P*_H2O2_ (nmol cell^−1^ h^−1^) was then calculated with the equation:

(9) $$P_{H2O2}=\frac{{\left[H_{2}O_{2}\right]}_{cell}\times Q}{N}$$

where *Q* is the flow rate (L h^−1^) and *N* is the number of cells on the filter, calculated from the measured cell density in the experimental culture and the volume of culture loaded onto the filter.

### Spiked batch incubations with spent culture medium

The ability of spent culture medium from *P. tricornutum* to produce and break down ROS was assessed by using spiked batch incubation methodology, as described in Vermilyea et al. (2010). Briefly, mid-exponential *P. tricornutum* cultures were filtered using 0.22 μm PES syringe filters (Millipore) at approximately 10 mL min^−1^, changing the syringe filters after every 10 mL of culture. The filtrate was collected in two 60-mL syringes (Kendell Mono-ject), one of which was then spiked with additional H_2_O_2_ to determine whether simultaneous H_2_O_2_ decay took place. The syringes were then incubated at room temperature in the dark and [H_2_O_2_] was monitored over a period of several hours to determine production and decay rates.

If the production rate in the medium, *P'*_*H*2*O*2_ (nM h^−1^) is constant per unit volume, and decay is first-order with respect to [H_2_O_2_], the change in [H_2_O_2_] over time is given by the equation:

(10) $$\frac{d\left[H_{2}O_{2}\right]}{dt}=P_{H2O2}^{\prime }-k_{loss,H2O2}^{\prime }\left[H_{2}O_{2}\right]$$

where *k'*_*loss, H*2*O*2_ (h^−1^) is the pseudo-first order rate coefficient of H_2_O_2_ decay. Time and concentration data from each spiked batch incubation were fitted to the solution to differential Equation 10:

(11) $$\left[H_{2}O_{2}\right]=\frac{P_{H2O2}^{\prime }}{k_{loss,H2O2}^{\prime }}-\left\{\left(\frac{P_{H2O2}^{\prime }}{k_{loss,H2O2}^{\prime }}-{\left[H_{2}O_{2}\right]}_{0}\right)e^{-k_{loss,H2O2^{t}}^{\prime }}\right\}$$

using the Microsoft Excel Solver function with the initial [H_2_O_2_], [H_2_O_2_]_0_, of each incubation and global *P*${}_{H2O2}^{\prime }$ and *k'*_*loss, H*2*O*2_ values for both incubations as fitting parameters.

### Replicates and measurement uncertainties

Dark and light live production rates for O${}_{2}^{-}$ and H_2_O_2_ were measured in duplicate on a given experimental day, while single measurements of killed dark and light production rates were measured each day. Data for each condition from two separate experimental cultures, examined on different days, were pooled for each organism except as noted.

Statistical significance for all results was assessed using a two-tailed *t*-test with the minimum level for significance at *p* = 0.05. Reported uncertainties in figures and text represent one standard deviation. Detection limits for P_O2_ and P_H2O2_ are a function of the size of the analytical and background signals, the recovery, and the cell density, and therefore varied from run to run. For the purposes of this work, we treat any set of replicate results whose average was not significantly greater than zero (using a *t*-test and *p* = 0.05) as below detection limit.

## Results

### *Thalassiosira* culture ROS production

#### O${}_{2}^{-}$

**Figure 1:**
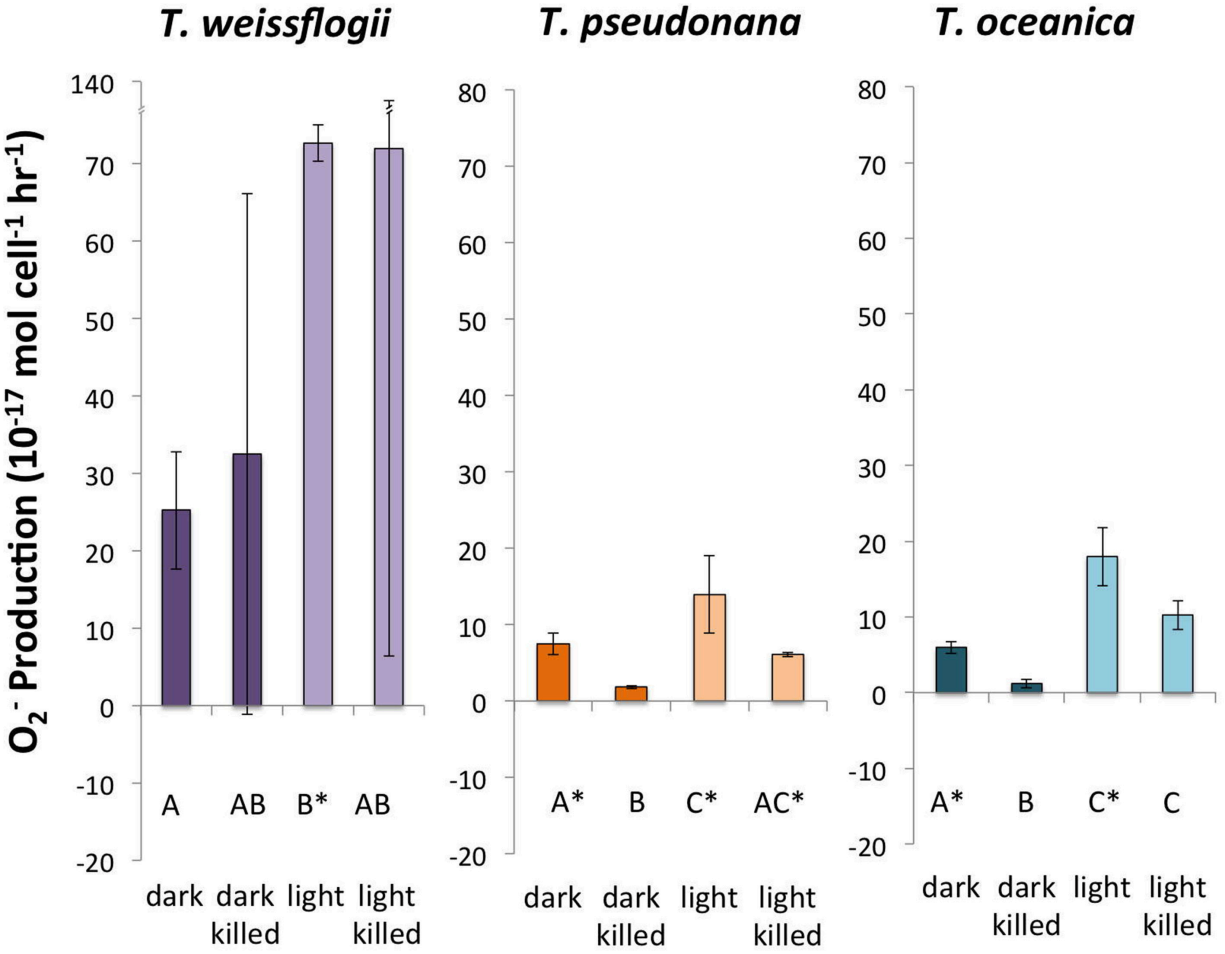
**Calculated O${}_{2}^{-}$ production rates in genus *Thalassiosira***. Error bars represent one standard deviation. Letters designate rates significantly different from each other within each panel. Asterisks designate P_O2−_ values significantly different from zero.

Successful measurements of P_O2−_ were performed in cultures of all three *Thalassiosira* spp. under all four conditions (Figure 1). *T. oceanica* and *T. pseudonana* had similar live P_O2−_, with both dark and light live conditions giving production rates significantly greater than zero. *T. oceanica* had an average P_O2−_ of 18.0 ± 3.9 × 10^−17^ mol cell^−1^ h^−1^ in the light and 6.0 ± 0.8 × 10^−17^ mol cell^−1^ h^−1^ in the dark (*n* = 4). *T. pseudonana* had an average P_O2−_ of 13.9 ± 5.1 × 10^−17^ mol cell^−1^ h^−1^ in the light and 7.5 ± 1.5 × 10^−17^ mol cell^−1^ h^−1^ in the dark (*n* = 4). For both of these organisms, the live P_O2−_ values were also considerably greater (~2–5 times) than the corresponding killed production rates, with the difference being statistically significant for the dark values. *T. weissflogii* had much greater production rates than the other two species, 72.7 ± 2.3 × 10^−17^ mol cell^−1^ h^−1^ in the light and 25.2 ± 7.6 × 10^−17^ mol cell^−1^ h^−1^ in the dark (*n* = 2). However, *T. weissflogii* is also the largest of the three organisms, and its production rates look similar to those of *T. oceanica* and *T. pseudonana* when normalized to surface area (Section S4, Figure S6). Because of the lower number of replicates for *T. weissflogii* analyses, the measurement uncertainties for this species were high (the spike signal was undetectable in the replicate analysis, so Rec_*O*2−_ and P_O2−_ could not be determined). As a result, only the light live P_O2−_ is significantly greater than zero for this organism.

**Figure 2:**
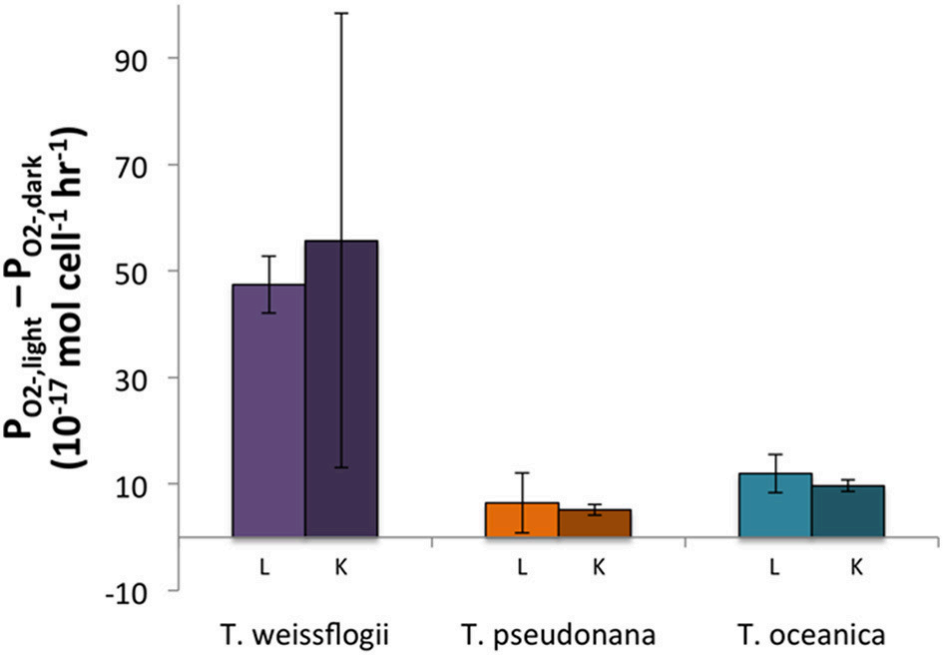
**Differences between light and dark P_O2−_ for both live (L) and killed (K) cultures in *Thalassiosira* spp**. Error bars represent one standard deviation.

The light treatments had a greater P_O2−_ than the dark treatments (~2–8 times more) for all three organisms, and the differences were statistically significant for both live and killed *T. oceanica* and *T. pseudonana*, and live *T. weissflogii*. The light-induced increase in live cultures did not differ significantly from the light-induced increase in killed cultures for any of the three organisms (Figure 2). These findings are summarized in Table 2.

**Table 2:** **Summary of light vs. dark and live vs. killed comparison for H_2_O_2_ and O${}_{2}^{-}$ production rates**.

	**H_2_O_2_ production**		**O${}_{2}^{-}$ production ratios**	
**Organism**	**Light > Dark?**	**Live > killed?**	**Light > dark?**	**Live > killed?**
*T. weissflogii*	NO^§^	NO^§^	YES^*^	NO
*T. pseudonana*	YES	YES	YES^*^	YES^*^
*T. oceanica*	YES^*^	YES^§^	YES^*^	YES^*^
*P. tricornutum*	YES	NO	N/A	N/A
*C. cryptica*	YES^§^	NO^§^	N/A	N/A

* *Indicates a statistically significant comparison*.

§ *Indicates a comparison in which at least one of the production values is negative*.

#### H_2_O_2_

**Figure 3:**
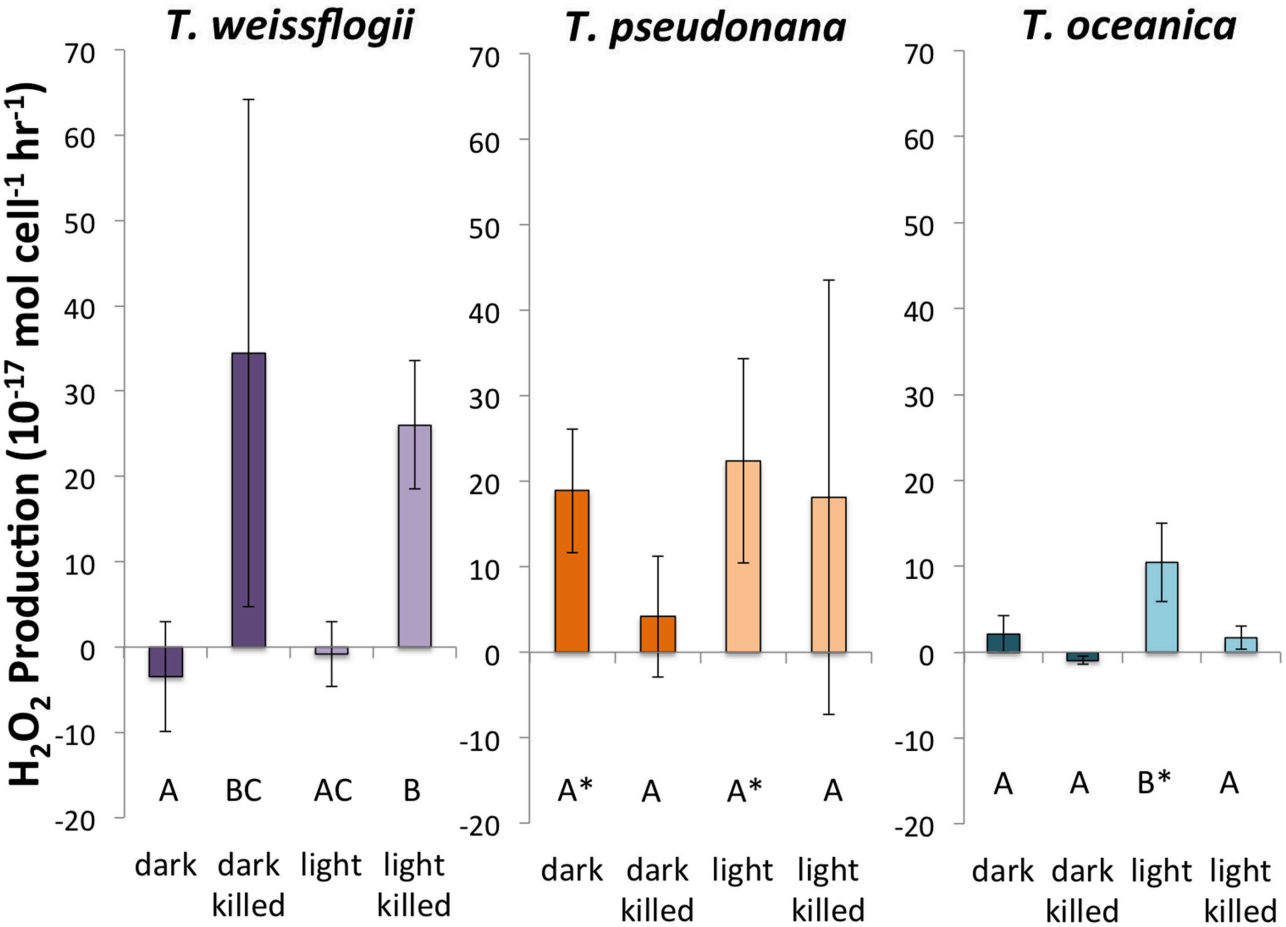
**Calculated H_2_O_2_ production rates in *Thalassiosira* spp**. Error bars represent one standard deviation. Letters designate rates significantly different from each other within each panel. Asterisks represent H_2_O_2_ production rates that are significantly different from zero.

Of the three species of *Thalassiosira* studied, only live cultures of *T. oceanica* produced significantly more H_2_O_2_ in light than in dark conditions, 10.5 ± 4.6 × 10^−17^ mol cell^−1^ h^−1^ (*n* = 4) as opposed to 2.1 ± 2.1 × 10^−17^ mol cell^−1^ h^−1^ (*n* = 4) (Figure 3). *T. pseudonana* appeared to produce slightly, but not significantly, more H_2_O_2_ in light than in dark conditions: 22.4 ± 11.9 × 10^−17^ mol cell^−1^ h^−1^ (*n* = 4) vs. 18.9 ± 7.2 × 10^−17^ mol cell^−1^ h^−1^ (*n* = 4). By contrast, live cultures of *T. weissflogii* had H_2_O_2_ production rates statistically indistinguishable from zero in both dark (*n* = 3 with one statistical outlier, with a large negative production rate, removed) and light (*n* = 4) experiments. Killed cultures of both *T. pseudonana* and *T. oceanica* had H_2_O_2_ production rates indistinguishable from zero, whereas H_2_O_2_ production by killed cultures of *T. weissflogii* was relatively high, 34.5 ± 29.8 × 10^−17^ mol cell^−1^ h^−1^ in the dark (*n* = 2) and 26.1 ± 7.6 × 10^−17^ mol cell^−1^ h^−1^ in the light (Figure 3). Normalized to cell surface area, *T. pseudonana* produced much more H_2_O_2_ than the other two organisms (Section S4, Figure S7).

### ROS production in other axenic diatom cultures

#### O${}_{2}^{-}$

Live cultures of both *Phaeodactylum tricornutum* and *Cyclotella cryptica* were observed to produce a O${}_{2}^{-}$ signal that could be detected above the ASW medium. However, a P_O2−_ could not be calculated since Rec_*O*2_ could not be determined. For *P. tricornutum* the detection limit was 0.054 ± 0.024 nM or a Rec_*O*2−_ <12%. For *C. cryptica* the detection limit was 0.033 ± 0.012 nM or a Rec_*O*2−_ <5%.

#### H_2_O_2_

In both live and killed cultures of both organisms, H_2_O_2_ production rates were generally indistinguishable from zero (Section S3, Figure S5).

**Figure 4:**
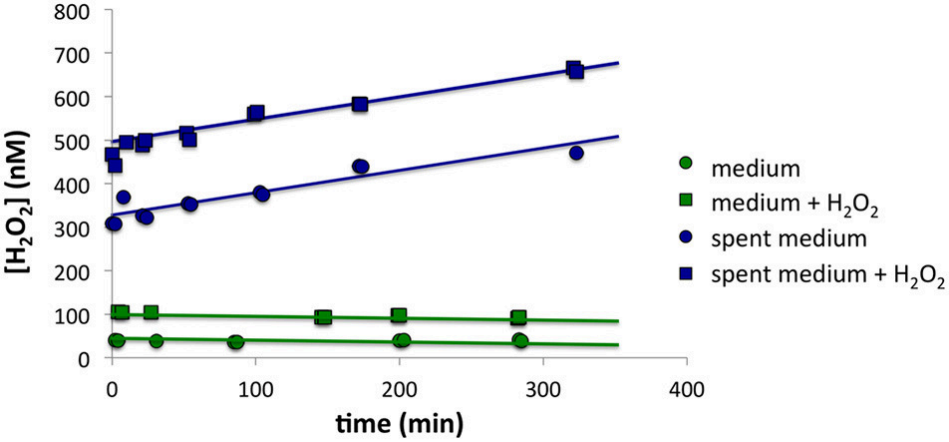
**Spiked batch incubations for (top) spent growth medium from *P. tricornutum* and (bottom) sterile F/2 medium**. The circles indicate unaltered medium, while the squares indicate medium that has been spiked with additional H_2_O_2_.

Although *P. tricornutum* cells did not appear to make much H_2_O_2_ on the filter, high [H_2_O_2_] in the growth medium was observed when cells were first loaded onto the filter, suggesting that the cells secreted ROS-producing small molecules or enzymes. Using the methodology described in the Section titled Spiked Batch Incubations with Spent Culture Medium, we observed that cell-free spent culture medium had a H_2_O_2_ decay rate below detection limit and an H_2_O_2_ production rate *P'*_*H*2*O*2_ that remained constant at 36.9 ± 9.1 nM h^−1^ (*n* = 2) over the course of 5 h. Taking into account the original cell densities, this would equate to a cell-normalized production rate, P_H2O2_, of 50 × 10^−17^ mol cell^−1^ h^−1^. By contrast, sterile F/2 medium had decay of 0.2 h^−1^ and production of 0.3 nM h^−1^ (*n* = 1) (Figure 4). Although high [O${}_{2}^{-}$] in the medium was also observed during cell loading, it was not possible to quantify O${}_{2}^{-}$ production rates in the spent medium with a similar methodology, due to the fast O${}_{2}^{-}$ decay rates.

### ROS recoveries

#### O${}_{2}^{-}$ recoveries

**Figure 5:**
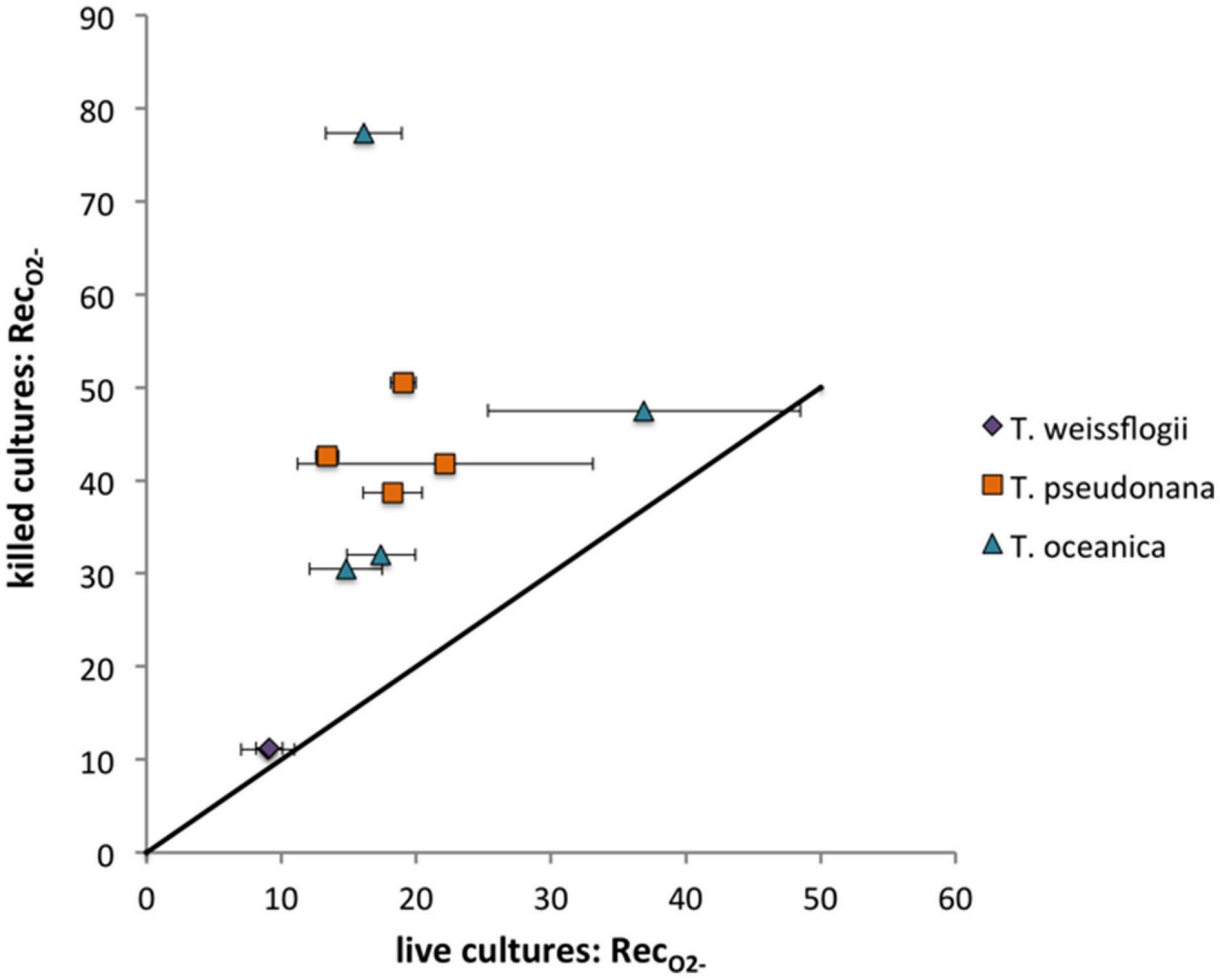
**O${}_{2}^{-}$ recoveries (%) for *Thalassiosira* cultures examined**. Live values shown are averages and standard deviations from duplicate measurements on the same day. The line represents a 1:1 relationship.

The O${}_{2}^{-}$ standard addition spike could be detected in all *Thalassiosira* cultures except for one of the replicate live *T. weissflogii* cultures. Each *Thalassiosira* species decreased the signal observed from the spike, with all the live cultures degrading significantly more O${}_{2}^{-}$ (indicated by lower Rec_*O*2−_) than the killed cultures (*P* < 0.05), except for *T. weissflogii*. The average live Rec_*O*2−_ were 18.2 ± 5.4% (*n* = 8), 21.3 ± 10.8% (*n* = 8), and 9.1 ± 1.3% (*n* = 4) for *T. pseudonana, T. oceanica*, and *T. weissflogii*, respectively. The average killed Rec_*O*2−_ were 43.4 ± 5.0% (*n* = 4), 46.8 ± 21.7% (*n* = 4), and 16.9 ± 6.9% (*n* = 4) for *T. pseudonana, T. oceanica*, and *T. weissflogii*, respectively. There were no significant differences in the Rec_*O*2−_ for the light and dark treatments for both *T. weissflogii* cultures and the live *T. pseudonana* cultures (*P* > 0.05) but differences were observed for killed *T. pseudonana* cultures and both *T. oceanica* cultures (*P* < 0.05) (Figure 5). Recovery values can be converted to surface-area-normalized O${}_{2}^{-}$ decay coefficients for a more direct comparison of these different organisms' ability to degrade O${}_{2}^{-}$. These calculations show that even on a surface-area normalized basis, *T. weissflogii* will degrade O${}_{2}^{-}$ more rapidly than the other two organisms (Section S5, Figure S8). The O${}_{2}^{-}$ spikes could not be detected in the presence of *C. cryptica* and *P. tricornutum*, meaning that Rec_*O*2−_ could not be determined.

#### H_2_O_2_ recoveries

**Figure 6:**
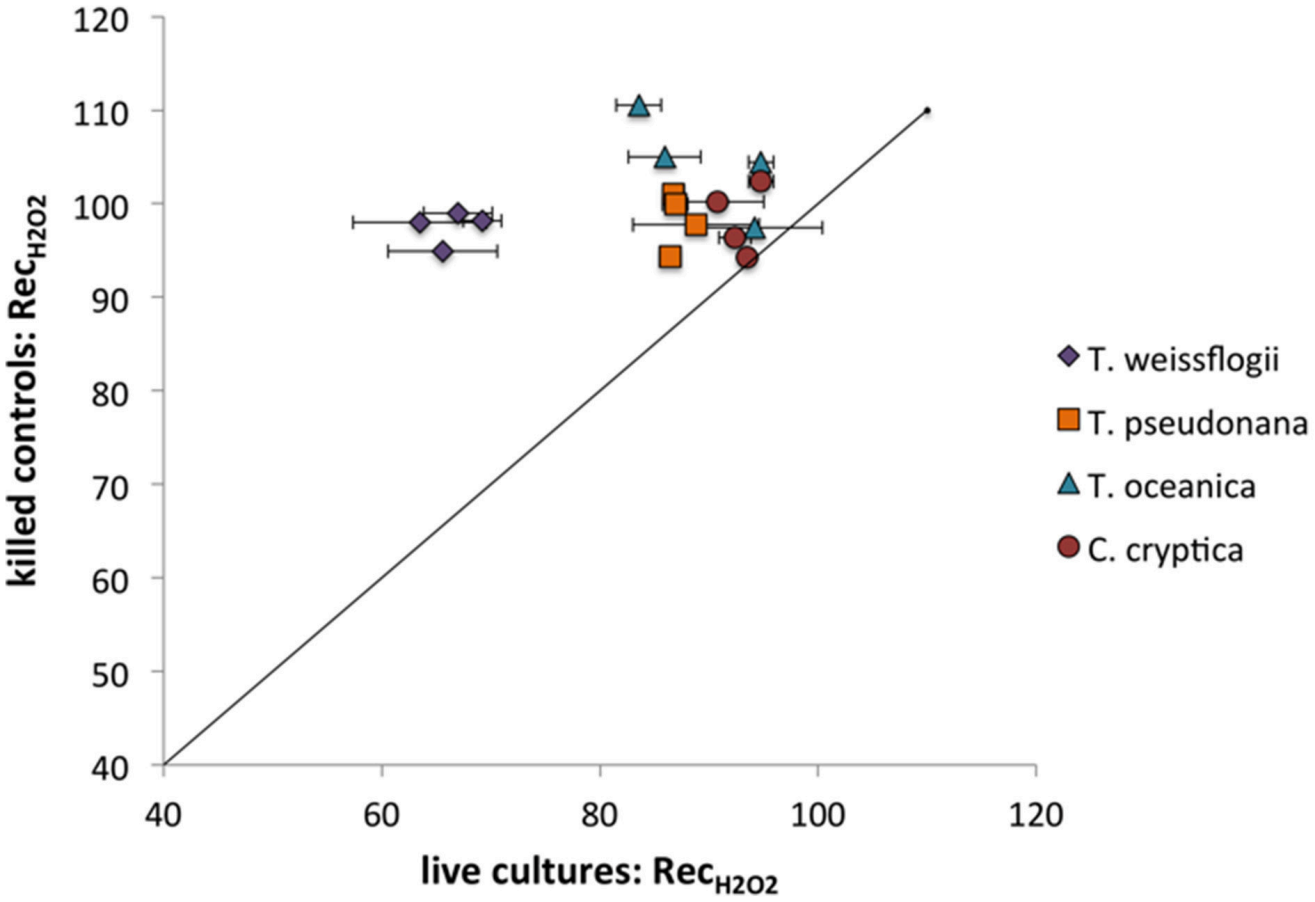
**H_2_O_2_ recovery percentages for live vs. killed organisms for *T. weissflogii*, *T. pseudonana*, *T. oceanica*, and *C. cryptica***. The line represents a 1:1 relationship.

All of the species in the present study decayed H_2_O_2_ (Figure 6), though the recoveries were always higher than for O${}_{2}^{-}$ (indicating less decay) under the same conditions (compare Figure 5 and Figure 6). Live *T. weissflogii* degraded significantly more H_2_O_2_ than the other organisms, with recovery rates of 66 ± 3% (*n* = 7), as opposed to rates ranging from 84 ± 5% in *P. tricornutum* (*n* = 8) to 93 ± 2% in *C. cryptica* (*n* = 8). Even on a cell-surface area normalized basis, *T. weissflogii*'s ability to degrade H_2_O_2_ was greater than those of the other organisms (Figure S9). H_2_O_2_ recoveries were statistically indistinguishable from 100% in killed organisms. Of the cultures studied, only *C. cryptica* had a value of Rec_*H*2*O*2_ for killed cultures (99 ± 5%, *n* = 4) that was not significantly different from that for live cultures. H_2_O_2_ recovery rates were not determined for killed cultures of *P. tricornutum*.

### H_2_O_2_ production in cultures loaded on the filter by syringe

**Figure 7:**
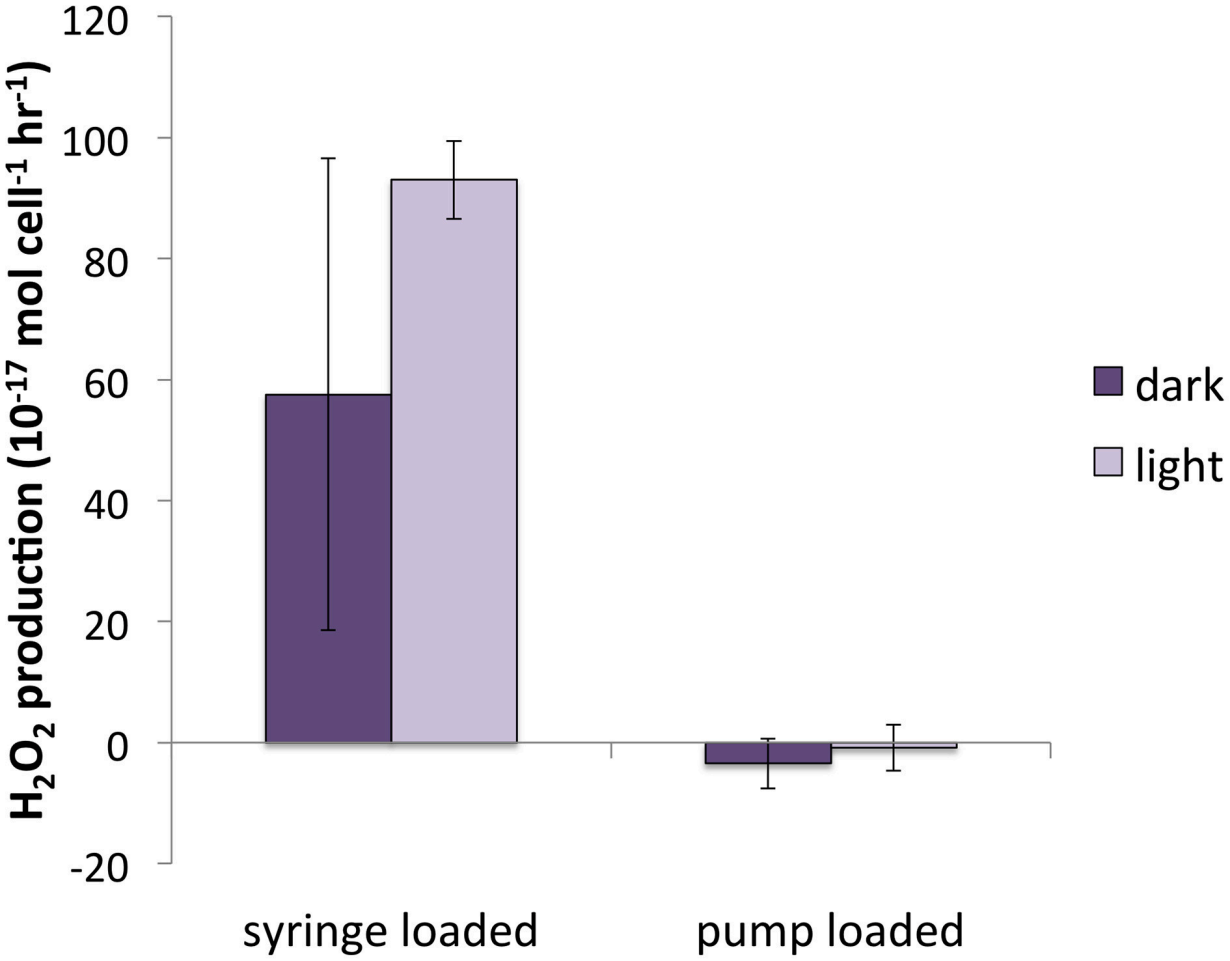
**H_2_O_2_ production for pump-loaded vs. syringe loaded cultures of *T. weissflogii***.

In early H_2_O_2_ experiments, aliquots of culture were loaded on the filter using a syringe. Differences between the syringe-loaded and pump-loaded live cultures were compared in two diatom species, *T. oceanica* and *T. weissflogii* (Figure 7). For purposes of this section, both light and dark measurements are pooled.

There was no significant difference between syringe-loaded cultures and pump-loaded cultures for *T. oceanica*. P_H2O2_ was 7.1 ± 5.5 × 10^−17^ mol cell^−1^ h^−1^ for pump-loaded cultures (*n* = 8) and 10.7 ± 4.0 × 10^−17^ mol cell^−1^ h^−1^ for syringe-loaded cultures (*n* = 9); recovery percentages were 88 ± 5 and 86 ± 3%, respectively. While recovery rates were also statistically indistinguishable for *T. weissflogii* (66 ± 4% and 63 ± 6% for pump-loaded cultures vs. syringe loaded cultures, respectively), production rates were substantially different: in pump-loaded cultures (*n* = 7), H_2_O_2_ production rates were statistically indistinguishable from zero, while in syringe-loaded cultures (*n* = 4), they were significantly higher at 80.3 ± 22.1 × 10^−17^ mol cell^−1^ h^−1^.

## Discussion

### Diatom O${}_{2}^{-}$ production

The O${}_{2}^{-}$ values reported in the present study compare well to the previously published values (Kustka et al., 2005; Rose et al., 2008; Milne et al., 2009) for *T. weissflogii* (Table 3). Both Milne et al. (2009) and the present study found that light enhances P_O2−_, though killed cultures were not examined in the former. It appears that the light response can also occur in low light since Milne et al. (2009) found increased P_O2−_ with as little as 30 μmol photons m^−2^ s^−1^ light intensity while 75 μmol photons m^−2^ s^−1^ was used in the present study. In the case of *T. pseudonana*, the present study reports values ~six times lower than Rose et al. (2008) who examined P_O2−_ under different conditions (Fe stress and different growth phases) than tested here.

**Table 3:** **Comparison of published diatom P_*O*2−_ and methods**.

	**Organism**	**P_*O*2−_(10^−17^ mol cell^−1^ h^−1^)**	**Culture treatment**	**O${}_{2}^{-}$ standard**	**% Recovery corrected**
Rose et al., 2008	*T. pseudonana*	40–83	A,B	X:XO	(Yes)—see Discussion
Schneider et al., (this study)	*T. pseudonana*	6–14	C	KO_2_	Yes
Kustka et al., 2005	*T. weissflogii*	84	A	Photolysis	No
Milne et al., 2009	*T. weissflogii*	25–132	D	X:XO	No
Rose et al., 2008	*T. weissflogii*	30–140	A,B	X:XO	(Yes)—see Discussion
Schneider et al., (this study)	*T. weissflogii*	25–73	C	KO_2_	Yes

Culture treatments are: A, Fe stress; B, different growth phases; C, normal conditions (0 or 75 μmol photons m^−2^ s^−1^); and D, light stress (30–500 μmol photons m^−2^ s^−1^). In O${}_{\text{2}}^{-}$ standards, X:XO stands for xanthine:xanthine oxidase.

Despite the similar results between the present study and the other three studies listed in Table 3, especially for *T. weissflogii*, there were several important methodological differences. Milne et al. (2009) and Rose et al. (2008) used the xanthine:xanthine oxidase system to generate O${}_{2}^{-}$ for calibration, while the present study used KO_2_ and Kustka et al. (2005) used photochemically generated O${}_{2}^{-}$ stock solutions. The poor stability of the xanthine:xanthine oxidase system, as well as its tendency to generate less O${}_{2}^{-}$ than the manufacturer's specifications of the enzyme's activity, have been noted in a previous study (Rose et al., 2008; Rose, 2012). Both Milne et al. (2009) and Rose et al. (2008) took these issues into account. However, Milne et al. (2009) also based their calibration on an assumed half-life of 100 s for O${}_{2}^{-}$ in seawater instead of directly measuring the half-life of O${}_{2}^{-}$ in their assay medium (a natural seawater sampled using a CTD). Because decay rates can vary widely depending on where the water was sampled (Rose et al., 2008, 2010; Hansard et al., 2010; Heller and Croot, 2010) and are also highly sensitive to trace metal contamination, the actual half-life of O${}_{2}^{-}$ could have been much lower than the value they assumed.

Another consideration is how the cells are influencing the O${}_{2}^{-}$ signal detected by the photomultiplier tube. The present study examined this effect using a known O${}_{2}^{-}$ spike and determined that the diatom cells which were immobilized on the filter (both live and killed) drastically reduced the O${}_{2}^{-}$ signal. Milne et al. (2009) and Kustka et al. (2005) also measured O${}_{2}^{-}$ production by cells immobilized on a filter, but did not correct the signal for this effect. In contrast, Rose et al. (2008) added MCLA directly to cell cultures and compared the resulting chemiluminescence signal to the signal from cultures with xanthine/xanthine oxidase added in addition to MCLA. While this technique did not explicitly measure “recovery” values, it would have automatically corrected for the effect of cells on the signal. However, their reported signal would have also included any O${}_{2}^{-}$ generated by cell exudates in the medium.

A final difference in these studies is the value assigned as the “baseline” signal. In Rose et al. (2008) the baseline was defined as a cell culture with MCLA and SOD added. Kustka et al. (2005) did not directly state what they used as a baseline signal, but implied that it is also the SOD-added signal. This means any background superoxide generated by the medium or the container would have also been included in both of these studies' reported measurements. Milne et al. (2009) used the signal measured in the absence of light as their baseline, so their reported values only measure the enhancement caused by light.

Given these substantial methodological differences, the correspondence of the results of previous studies with each other and the present study could be fortuitous. However, the present study is the only study that attempted to correct for both recovery effects and background signal.

### H_2_O_2_ production

A previous investigation of H_2_O_2_ production rates (Milne et al., 2009) by *T. weissflogii* found a range of P_H2O2_ between 11 and 66 × 10^−17^ mol cell^−1^ h^−1^ for cultures loaded onto the filter by syringe, under a range of light intensity (30–500 μmol photons m^−2^ s^−1^) and quantified by a similar method to that employed in the present study, but without the correction for simultaneous decay (Milne et al., 2009). P_H2O2_ values for syringe-loaded cultures in the present study were similar in magnitude (80.3 ± 22.1 × 10^−17^ mol cell^−1^ h^−1^); however, *T. weissflogii* cultures loaded onto the filter by peristaltic pump had P_H2O2_ indistinguishable from zero; these values were therefore significantly lower than those for syringe-loaded cultures. Even gentle syringe loading results in considerably higher flow rates (~5 mL min^−1^) than loading by peristaltic pump (0.6 mL min^−1^). We suspect that suspending cells on a filter under the higher pressure that accompanies syringe loading induced stress-related H_2_O_2_ production, which would suggest that *T. weissflogii* only produces substantial H_2_O_2_ under stress conditions.

A previous investigation of H_2_O_2_ production by *T. pseudonana* (Waring et al., 2010) used the Amplex Red™ method, which quantifies gross H_2_O_2_ production, and therefore those rates were expected to be comparable to the present study. Although production was not reported numerically, it can be inferred from Figure 3D in Waring that low-light production is 7 × 10^−7^ mol μg chl a^−1^ over a 30 min period. Given the value of 4.45 × 10^−8^ μg chl a cell^−1^ (Table 2 in Waring), we calculated a much higher value of P_H2O2_ than seen in the present study, 7 × 10^−14^ mol cell^−1^ h^−1^. However, this discrepancy is the result of a calculation error (J. Waring, pers. comm.). The corrected H_2_O_2_ production rate is three orders of magnitude lower, meaning that their calculated value is not statistically different from the P_H2O2_ measured under light conditions in the present study.

H_2_O_2_ production by the other three species—*T. oceanica, C. cryptica*, and *P. tricornutum*—has not previously been studied.

### O${}_{2}^{-}$ and H_2_O_2_ recoveries

O${}_{2}^{-}$ recoveries in the presence of cells have only been quantified in one previous study. Diaz et al. (2013) observed that heterotrophic bacteria exhibited a range of O${}_{2}^{-}$ recovery, with values ranging from 1 to 100% depending on the bacterial species. By contrast, the present study shows that diatoms fall at the low end of this scale, with recoveries for live organisms ranging from < 5 to 50%. While the low O${}_{2}^{-}$ recoveries in the present study suggest that O${}_{2}^{-}$ decay by phytoplankton could potentially contribute to O${}_{2}^{-}$ decay in the environment, a recent field study saw no significant effect of filtering on decay rates in water samples from Station ALOHA and the California Current (Roe et al., 2016).

The low recoveries shown in the present study indicate that phytoplankton break down H_2_O_2_, which is consistent with previous findings (Wong et al., 2003). In addition, Wong et al. found that neither killed phytoplankton nor spent cell medium decayed H_2_O_2_. This aligns with the findings of the present study, in which recovery percentages for H_2_O_2_ were generally 100% for killed organisms, and in which very little decay occurred in spent growth medium from *P. tricornutum*. Overall this suggests that H_2_O_2_ decay occurs through an active cell process, whereby organisms ultimately control the H_2_O_2_ levels in their vicinity. Interestingly, H_2_O_2_ decay is not necessarily an absolute characteristic of all microorganisms; in fact, some symbiotic relationships between microbial consortia may be based, in part, on the need for catalase-deficient hosts to acquire H_2_O_2_-degrading symbionts (Morris et al., 2011).

While active cell processes seemed to be responsible for degradation of H_2_O_2_, the same cannot be said for decay of O${}_{2}^{-}$. Although recovery rates for killed organisms were higher in live organisms (Figure 5), in no case did they ever reach 100%. Thus, while decomposition of O${}_{2}^{-}$ is likely to be mediated in part by active cell processes, at least some of it occurs through a passive process.

### Controls on biological ROS production

Live *T. pseudonana* and *T. oceanica* appear to be actively producing O${}_{2}^{-}$ in the dark, since killing these organisms results in a significant decrease in P_O2−_ (results for *T. weissflogii* are not definitive because of the uncertainties in the measured P_O2−_ values for this species) (Table 2). This dark production corresponds with previous studies that link P_O2−_ to light-independent activity of outer membrane and/or transmembrane NADPH oxidases (Kim et al., 2000; Kustka et al., 2005; Saragosti et al., 2010). Similar transmembrane NADPH oxidases are known to mediate a number of essential physiological processes, such as cell development, signaling, and defense, in various eukaryotes, including fungi, plants, and mammalian cells (Saran, 2003; Bedard et al., 2007; Tsukagoshi et al., 2010). The potential for a physiological role for superoxide in diatoms, including *Thalassiosira*, is currently unknown and requires further exploration.

As observed previously (Kim et al., 2004; Kustka et al., 2005; Saragosti et al., 2010), P_O2−_ was enhanced for all three *Thalassiosira* species in the presence of light (Table 2). However, the magnitude of the enhancement appeared similar in live and killed cultures (Figure 2), so a passive photochemical source such as photo-oxidation of pigments present in the cell membrane cannot be ruled out as the additional source of superoxide. However, this is somewhat surprising given the low light levels used.

**Figure 8:**
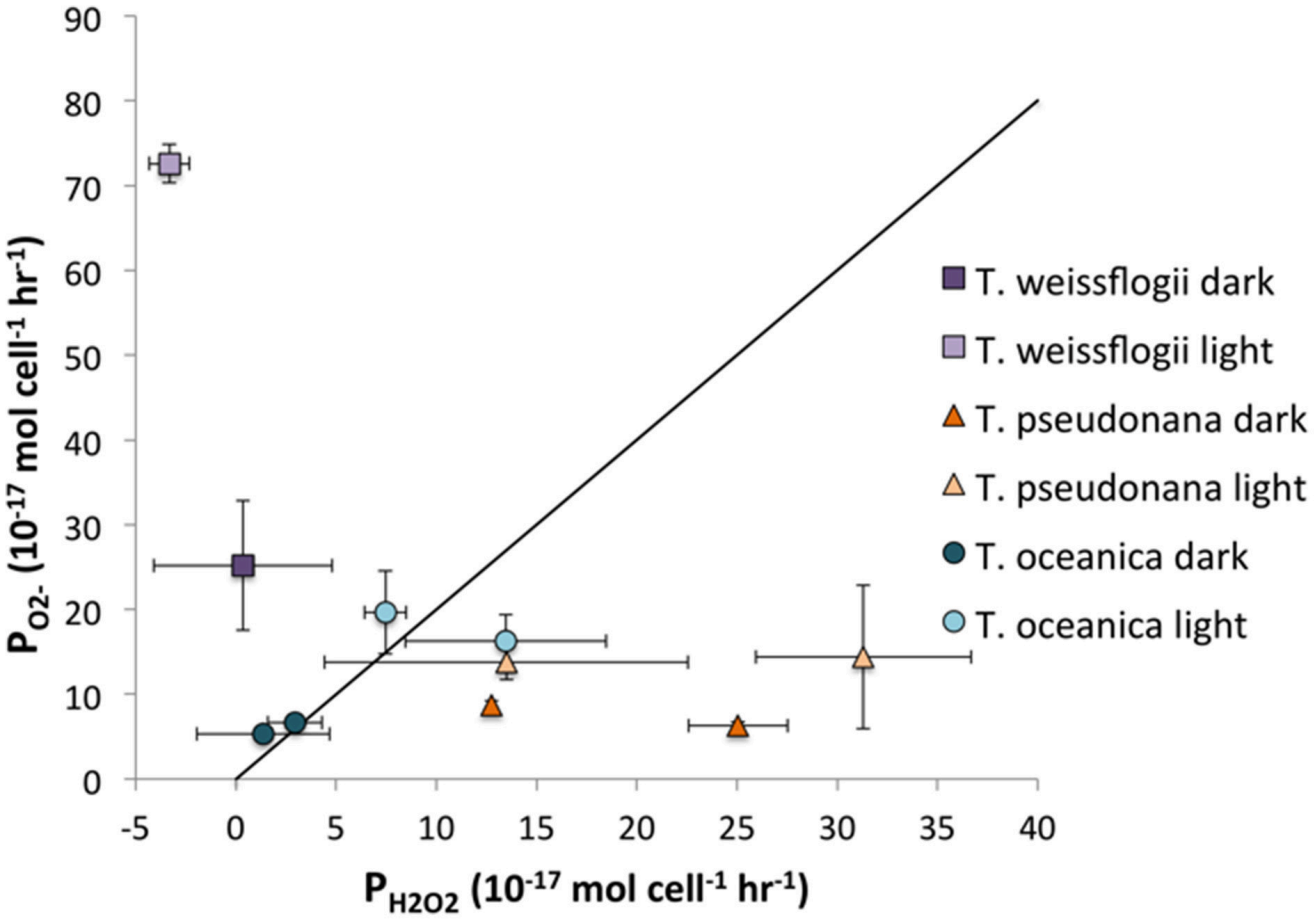
**Representation of the P_O2−_:P_H2O2_ ratio**. The diagonal line indicates the 2:1 ratio that would be obtained if all H_2_O_2_ was produced through dismutation of O${}_{2}^{-}$.

For the *Thalassiosira* spp, it is possible to compare the ratio of P_H2O2_ to P_O2−_ to determine how much, if any, of the H_2_O_2_ could be produced via O${}_{2}^{-}$. The ratio of H_2_O_2_ formation from O${}_{2}^{-}$ can vary anywhere from 0, if all of the O${}_{2}^{-}$ is oxidized, to 0.5, if dismutation occurs, to 1, if all of the O${}_{2}^{-}$ is reduced. The relationship between P_O2−_ and P_H2O2_ is shown in Figure 8. For *T. oceanica*, the data cluster around the 2:1 P_O2−_:P_H2O2_ line, suggesting that most or all of the H_2_O_2_ is produced via O${}_{2}^{-}$ dismutation. By contrast, P_H2O2_:P_O2−_ data for *T. pseudonana* plot far under the same line, signifying H_2_O_2_ production in excess of what could be produced from O${}_{2}^{-}$ dismutation, or even reduction, alone.

One source of this surplus H_2_O_2_ might be diffusion of intracellular H_2_O_2_ through the cell membrane. H_2_O_2_ can diffuse across the lipid bilayer to a small extent (Seaver and Imlay, 2001), but a larger quantity simply passes through aquaporins (Halliwell and Gutteridge, 2007). Extracellular H_2_O_2_ production is often thought to represent cell leakage of photosynthetically reduced oxygen under high light conditions (Suggett et al., 2008; Waring et al., 2010). In the absence of light, leakage of intracellular hydrogen peroxide formed from oxygen reduction at other electron transport chains is also probable (Forman and and Boveris, 1982). Indeed, most H_2_O_2_ production by *C. marina* is believed to originate from intracellular processes, which is magnified under cell stress and loss of membrane integrity (Kim et al., 2007). However, H_2_O_2_ can also be produced extracellularly. A previous study found that proteins on the cell surface of the coccolithophore *P. carterae* produced H_2_O_2_ without simultaneously producing O${}_{2}^{-}$ (Palenik et al., 1987) using a two-electron reduction of oxygen for nitrogen metabolism (Palenik and Morel, 1990). At present it is not clear whether the excess H_2_O_2_ produced by *T. pseudonana* originates from extracellular and/or intracellular sources.

By contrast with *T. pseudonana, T. weissflogii* produces far more than twice as much O${}_{2}^{-}$ as H_2_O_2_, indicating that not all O${}_{2}^{-}$ undergoes dismutation to form H_2_O_2_ but rather is destroyed by a different process, e.g., oxidation by organic matter. Alternatively, the higher flow rates in the O${}_{2}^{-}$ determination as compared to those in the H_2_O_2_ experiment might lead to higher ROS production overall; the present study clearly shows that *T. weissflogii* cells produce more H_2_O_2_ when subjected to higher flow rates, and this may also be true for O${}_{2}^{-}$ production.

This study shows that diatoms have a wide range of values for P_H2O2_ that hint at a diversity of biological pathways involved in production. Neither *C. cryptica* nor *P. tricornutum* produced measurable extracellular H_2_O_2_ under the tested conditions. Likewise, we cannot rule out that they produce O${}_{2}^{-}$, but if they do, they decompose it so effectively that the present study could not quantify it. While significant production of ROS was not detected from *P. tricornutum* cells, they do exude substances into the growth medium that produce ROS. H_2_O_2_ production by cell exudates was not quantified for the other species because *P. tricornutum* is the only species for which unusually high ROS signals were observed during loading, when spent medium was passing through the filter. It is likely that *P. tricornutum* secretes enzymes that produce H_2_O_2_ and, possibly O${}_{2}^{-}$ (though the present study was unable to test the latter). In fact, secretion of extracellular enzymes that produce O${}_{2}^{-}$ has previously been observed for *C. marina* (Kim et al., 2000) and a bacteria within the Roseobacter clade (Learman et al., 2011). These enzymes have been identified as an animal heme peroxidase for the Roseobacter bacterium (Andeer et al., 2015) and a protein analogous to the neutrophil NADPH oxidase in *C.* marina (Kim et al., 2000).

## Concluding remarks

Previous field studies (Rose et al., 2008; Vermilyea et al., 2010; Roe et al., 2016) showed that dark biological production of ROS is significant in comparison with photochemical production. Assuming all large phytoplankton, such as diatoms, have ROS production rates of the same approximate magnitude as those in this study, and assuming cell counts of 10^4^ (in oligotrophic waters such as Station ALOHA; Venrick, 1997) to 10^6^ (in the Gulf of Alaska; Paul et al., 1991) cells L^−1^, the phytoplankton contribution to this dark biological production would be 1–100 pM h^−1^, a small fraction of the observed 1–8 nM h^−1^. It is likely that dark biological ROS production is usually driven by the more numerous cyanobacteria (as in Rose et al., 2008) and heterotrophic bacteria (Diaz et al., 2013). However, phytoplankton such as diatoms may make major contributions to steady state concentrations of O${}_{2}^{-}$ and H_2_O_2_ during blooms, when their abundance can increase 10-fold (Villareal et al., 2012); this corresponds to observations showing higher O${}_{2}^{-}$ concentrations in *Trichodesmium* blooms (Rose et al., 2010).

Although diatoms may not be the primary influence on ROS concentrations in the ocean, their ROS production is likely an essential physiological process. In fact, previous studies have shown that O${}_{2}^{-}$ production is linked to Fe uptake in *Trichodesmium* (Roe and Barbeau, 2014) and *Lyngbya majuscula* (Rose et al., 2008) and cell growth/signaling in *C. marina* (Oda et al., 1995; Marshall et al., 2005), and that H_2_O_2_ may be a byproduct of nitrogen metabolism in *P. carterae* (Palenik et al., 1987). Given that similar species of diatoms produce ROS via different pathways, it is likely a suite of physiological benefits are conferred through ROS production that vary with species and, likely, environmental conditions.

## Author contributions

BV: Directed the research. RS: Designed experiments, cultured algae, conducted hydrogen peroxide measurements. KR: Designed experiments, cultured algae, conducted superoxide measurements. CH: Aided in design and interpretation of experiments.

### Conflict of interest statement

The authors declare that the research was conducted in the absence of any commercial or financial relationships that could be construed as a potential conflict of interest.

## References

[B1] Andeer P. F. Learman D. R. McIlvin M. Dunn J. A. Hansel C. M. (2015). Extracellular heme peroxidases mediate Mn (II) oxidation in a marine *Roseobacter* bacterium via superoxide production. Environ. Microbiol. 17, 3925–3936. 10.1111/1462-2920.12893 25923595

[B2] Avery G. B. Jr. Cooper W. J. Kieber R. J. Willey J. D. (2005). Hydrogen peroxide at the Bermuda Atlantic Time Series Station: temporal variability of seawater hydrogen peroxide. Mar. Chem. 97, 236–244. 10.1016/j.marchem.2005.03.006

[B3] Bedard K. Lardy B. Krause K. H. (2007). NOX family NADPH oxidases: not just in mammals. Biochimie 89, 1107–1112. 10.1016/j.biochi.2007.01.012 17400358

[B4] Cooper W. J. Moegling J. K. Kieber R. J. Kiddle J. J. (2000). A chemiluminescence method for the analysis of H_2_O_2_ in natural waters. Mar. Chem. 70, 191–200. 10.1063/1.555739

[B5] Cooper W. J. Zika R. G. (1983). Photochemical formation of hydrogen peroxide in surface and ground waters exposed to sunlight. Science 220, 711–712. 17813875 10.1126/science.220.4598.711

[B6] Cooper W. J. Zika R. G. Petasne R. G. Plane J. M. C. (1988). Photochemical formation of H_2_O_2_ in natural waters exposed to sunlight. Environ. Sci. Technol. 22, 1156–1160. 22148607 10.1021/es00175a004

[B7] Diaz J. M. Hansel C. M. Voelker B. M. Mendes C. M. Andeer P. F. Zhang T. (2013). Widespread production of extracellular superoxide by heterotrophic bacteria. Science 340, 1223–1226. 10.1126/science.1237331 23641059

[B8] Forman H. J. and Boveris A. (1982). Superoxide radical and hydrogen peroxide in mitochondria, in Free radicals in biology, ed Pryor W. (New York, NY: Academic) 65–90.

[B9] Godrant A. Rose A. L. Sarthou G. Waite T. D. (2009). New method for the determination of extracellular production of superoxide by marine phytoplankton using the chemiluminescence probes MCLA and red-CLA. Limnol. Oceanogr. Meth. 7, 682–692. 10.4319/lom.2009.7.682

[B10] Goldstone J. V. Voelker B. M. (2000). Chemistry of superoxide radical in seawater: CDOM associated sink of superoxide in coastal waters. Environ. Sci. Technol. 34, 1043–1048. 10.1021/es9905445

[B11] Halliwell B. Gutteridge J. M. C. (2007). Free Radicals in Biology and Medicine, 4 Edn. Oxford: Oxford University Press.

[B12] Hansard S. P. Vermilyea A. W. Voelker B. M. (2010). Measurements of superoxide radical concentration and decay kinetics in the Gulf of Alaska. Deep Sea Res. Pt. I 57, 1111–1119. 10.1016/j.dsr.2010.05.007

[B13] Heller M. I. Croot P. L. (2010). Superoxide decay kinetics in the southern ocean. Environ. Sci. Technol. 44, 191–196. 10.1021/es901766r 20039749

[B14] Herut B. Shoham-Frider E. Kress N. Angel D. (1998). Hydrogen peroxide production rates in clean and polluted coastal marinewaters of the mediterranean, red and baltic seas. Mar. Pollut. Bull. 36, 994–1003.

[B15] Kawano I. Oda T. Ishimatsu A. Muramatsu T. (1996). Inhibitory effects of the iron chelator desferrioxamine (Desferal) on the generation of activated oxygen spcies of *Chattonella marina*. Mar. Biol. 126, 765–771. 10.1007/BF00351343

[B16] Kim D. Nakamura A. Okamoto T. Komatsu N. Oda T. Iida T.. (2000). Mechanism of superoxide anion generation in the toxic red tide phytoplankton *Chattonella marina*: possible involvement of NAD(P)H oxidase. BBA 1524, 1–8. 10.1016/s0304-4165(00)00161-6 11113571

[B17] Kim D. Nakamura A. Okamoto T. Komatsu N. Oda T. Ishimatsu A. (1999). Toxic potential of the raphidophyte *Olisthodiscus luteus:* mediation by reactive oxygen species. J. Plankton Res. 21, 1017–1027.

[B18] Kim D. Nakashima T. Matsuyama Y. Niwano Y. Yamaguchi K. Oda T. (2007). Presence of the distinct systems responsible for superoxide anion and hydrogen peroxide generation in red tide phytoplankton *Chattonella marina* and *Chattonella ovata*. J. Plankton Res. 29, 241–247. 10.1093/plankt/fbm011

[B19] Kim D. Watanabe M. Nakayasu Y. Kohata K. (2004). Production of superoxide anion and hydrogen peroxide associated with cell growth of Chattonella antiqua. Aquat. Microb. Ecol. 35, 57–64. 10.3354/ame035057

[B20] King D. W. Cooper W. J. Rusak S. A. Peake B. M. Kiddle J. J. O'sullivan D. W. (2007). Flow injection analysis of H 2O 2in natural waters using acridinium ester chemiluminescence: method development and optimization using a kinetic model. Anal. Chem. 79, 4169–4176. 10.1021/ac062228w 17455905

[B21] Kustka Adam, B. Shaked Y. Milligan A. J. King D. W. Morel F. M. M. (2005). Extracellular production of superoxide by marine diatoms: contrasting effects on iron redox chemistry and bioavailability. Limnol. Oceanogr. 50, 1172–1180. 10.4319/lo.2005.50.4.1172

[B22] Learman D. R. Voelker B. M. Vazquez-Rodriguez A. I. Hansel C. M. (2011). Formation of manganese oxides by bacterially generated superoxide. Nat. Geosci. 4, 95–98. 10.1038/ngeo1055

[B23] Marshall J.-A. Hovenden M. Oda T. Hallegraeff G. M. (2002). SHORT COMMUNICATION Photosynthesis does influence superoxide production in the ichthyotoxic alga *Chattonella marina* (*Raphidophyceae*). J. Plankton Res. 24, 1231–1236. 10.1093/plankt/24.11.1231

[B24] Marshall J. A. Ross T. Pyecroft S. Hallegraeff G. (2005). Superoxide production by marine microalgae—II. Towards understanding ecological consequences and possible functions. Mar. Biol. 147, 541–549. 10.1007/s00227-005-1597-6

[B25] Miller W. L. Kester D. R. (1988). Hydrogen peroxide measurement in seawater by (para-hydroxylphenyl) acetic acid dimerization. Anal. Chem. 60, 2711–2715.

[B26] Milne A. Davey M. S. Worsfold P. J. Achterberg E. P. Taylor A. R. (2009). Real-time detection of reactive oxygen species generation by marine phytoplankton using flow injection–chemiluminescence. Limnol. Oceanogr. 7, 706–715. 10.4319/lom.2009.7.706

[B27] Moffett J. W. Zafiriou O. C. (1990). An investigation of hydrogen peroxide chemistry in surface waters of Vineyard Sound with H${}_{2}^{18}$O_2_ and ^18^O_2_. Limnol Oceanogr. 35, 1221–1229.

[B28] Morris J. J. Johnson Z. I. Szul M. J. Keller M. Zinser E. R. (2011). Dependence of the cyanobacterium *Prochlorococcus* on hydrogen peroxide scavenging microbes for growth at the ocean's surface. PLoS ONE 6:e16805. 10.1371/journal.pone.0016805 21304826 PMC3033426

[B29] Oda T. Moritomi J. Kawano I. Hamaguchi S. Ishimatsu A. Muramatsu T. (1995). Catalase- and superoxide dismutase-induced morphological changes and growth inhibition in the red tide phytoplankton *Chattonella marina*. Biosci. Biotech. Biochem. 59, 2044–2048.

[B30] Oda T. Nakamura A. Shikayama M. Kawano I. Ishimatsu A. Muramatsu T. (1997). Generation of reactive oxygen species by raphidophycean phytoplankton. Biosci. Biotechnol. Biochem. 61, 1658–1662. 9362113 10.1271/bbb.61.1658

[B31] Palenik B. Morel F. M. M. (1990). Amino acid utilization by marine phytoplankton: a novel mechanism. Limnol. Oceanogr. 35, 260–269. 10.4319/lo.1990.35.2.0260

[B32] Palenik B. Zafiriou O. C. Morel F. M. M. (1987). Hydrogen peroxide production by a marine phytoplankter. Limnol. Oceanogr. 32, 1365–1369.

[B33] Paul A. J. Paul J. M. Coyle K. Smith R. (1991). Phytoplankton, Zooplankton, and Ichthyoplankton in Resurrection Bay, Northern Gulf of Alaska in 1988. Fairbanks, AK: Alaska Sea Grant College Program.

[B34] Petasne R. G. Zika R. G. (1997). Hydrogen peroxide lifetimes in south Florida coastal and offshore waters. Mar. Chem. 56, 215–225.

[B35] Price N. M. Harrison G. L. Hering J. G. Hudson R. J. Nirel P. M. V. Palenik B. (1989). Preparation and chemistry of the artificial algal culture medium aquil. Biol. Oceanogr. 6, 443–461. 10.1080/01965581.1988.10749544

[B36] Roe K. L. Barbeau K. A. (2014). Uptake mechanisms for inorganic iron and ferric citrate in *Trichodesmium erythraeum* IMS 101. Metallomics 6, 2042–2051. 10.1039/C4MT00026A 25222699

[B37] Roe K. L. Schneider R. J. Hansel C. M. Voelker B. M. (2016). Measurement of dark, particle-generated superoxide and hydrogen peroxide in the subtropical and temperate North Pacific Ocean. Deep Sea Res. Pt I 107, 59–69. 10.1016/j.dsr.2015.10.012

[B38] Rose A. L. (2012). The influence of extracellular superoxide on iron redox chemistry and bioavailability to aquatic microorganisms. Front. Microbiol. 124, 1–21. 10.3389/fmicb.2012.00124 22514548 PMC3323869

[B39] Rose A. L. Godrant A. Furnas M. Waite T. D. (2010). Dynamics of nonphotochemical superoxide production in the Great Barrier Reef lagoon. Limnol. Oceanogr. 55, 1521–1536. 10.4319/lo.2010.55.4.1521

[B40] Rose A. L. Moffett J. W. Waite T. D. (2008). Determination of superoxide in seawater using 2-methyl-6-(4-methoxyphenyl)-3,7-dihydroimidazo[1,2-a]pyrazin-3(*7H*)-one chemiluminescence. Anal. Chem. 80, 1215–1227. 10.1021/ac7018975 18201070

[B41] Rose A. L. Salmon T. P. Lukondeh T. Neilan B. A. Waite T. D. (2005). Use of superoxide as an electron shuttle for iron acquisition by the marine cyanobacterium *Lyngbya majuscula*. Environ. Sci. Technol. 39, 3708–3715. 10.1021/es048766c 15952376

[B42] Rose A. L. Webb E. A. Waite T. D. Moffett J. W. (2008). Measurement and implications of nonphotochemically generated superoxide in the equatorial Pacific ocean. Environ. Sci. Technol. 42, 2387–2393. 10.1021/es7024609 18504970

[B43] Saito M. A. Moffett J. W. Chisholm S. W. Waterbury J. B. (2002). Cobalt limitation and uptake in *Prochlorococcus*. Limnol. Oceanogr. 47, 1629–1636. 10.4319/lo.2002.47.6.1629

[B44] Saragosti E. Tchernov D. Katsir A. Shaked Y. (2010). Extracellular production and degradation of superoxide in the coral *Stylophora pistillata* and cultured *Symbiodinium*. PLoS ONE 5:e12508. 10.1371/journal.pone.0012508 20856857 PMC2939047

[B45] Saran M. (2003). To what end does nature produce superoxide? NADPH oxidase as an autocrine modifier of membrane phospholipids generating paracrine lipid messengers. Free Radic. Res. 37, 1045–1059. 10.1080/10715760310001594631 14703794

[B46] Scholz W. Galvan F. de la Rosa F. F. (1995). The microalga *Chlamydomonas reinhardtii* CW-15 as a solar cell for hydrogen peroxide photoproduction: comparison between free and immobilized cells and thylakoids for energy conversion efficiency. Sol. Ener. Mater. Sol. Cells 39, 61–69.

[B47] Seaver L. C. Imlay J. A. (2001). Hydrogen peroxide fluxes and compartmentalization inside growing *Escherichia coli*. J. Bacteriol. 183, 7182–7189. 10.1128/JB.183.24.7182-7189.2001 11717277 PMC95567

[B48] Shaked Y. Harris R. Klein-Kedem N. (2010). Hydrogen peroxide photocycling in the gulf of aqaba, red sea. Environ. Sci. Technol. 44, 3238–3244. 10.1021/es902343y 20377174

[B49] Suggett D. J. Warner M. E. Smith D. J. Davey P. Hennige S. Baker N. R. (2008). Photosynthesis and production of hydrogen peroxide by Symbiodinium (Pyrrhophyta) phylotypes with different thermal tolerances. J. Phycol. 44, 948–956. 10.1111/j.1529-8817.2008.00537.x 27041613

[B50] Tsukagoshi H. Busch W. Benfey P. N. (2010). Transcriptional regulation of ROS controls transition from proliferation to differentiation in the root. Cell 143, 606–616. 10.1016/j.cell.2010.10.020 21074051

[B51] Twiner M. J. Trick C. G. (2000). Possible physiological mechanisms for production of hydrogen peroxide by the ichthyotoxic flagellate Heterosigma akashiwo. J. Plankton Res. 22, 1961–1975. 10.1093/plankt/22.10.1961

[B52] Venrick E. L. (1997). Comparison of the phytoplankton species composition and structure in the Climax area (1973–1985) with that of station ALOHA (1994). Limnol. Oceanogr. 42, 1643–1648.

[B53] Vermilyea A. W. Hansard S. P. Voelker B. M. (2010). Dark production of hydrogen peroxide in the Gulf of Alaska. Limnol. Oceanogr. 55, 580–588. 10.4319/lo.2009.55.2.0580

[B54] Villareal T. A. Brown C. G. Brzezinski M. A. Krause J. W. Wilson C. (2012). Summer diatom blooms in the North Pacific subtropical gyre: 2008–2009. PLoS ONE 7, e33109–e33115. 10.1371/journal.pone.0033109 22493663 PMC3320889

[B55] Waring J. Klenell M. Bechtold U. Underwood G. J. C. Baker N. R. (2010). Light-induced responses of oxygen photoreduction, reactive oxygen species production and scavenging in two diatom species. J. Phycol. 46, 1206–1217. 10.1111/j.1529-8817.2010.00919.x

[B56] Wong G. T. F. Dunstan W. M. Kim D.-B. (2003). The decomposition of hydrogen peroxide by marine phytoplankton. Oceanol. Acta 26, 191–198. 10.1016/S0399-1784(02)00006-3

[B57] Wuttig K. Heller M. I. Croot P. L. (2013). Pathways of superoxide (O${}_{2}^{-}$) decay in the Eastern tropical North Atlantic. Environ. Sci. Technol. 47, 10249–10256. 10.1021/es401658t 23915117

[B58] Yamasaki Y. Kim D.-I. Matsuyama Y. Oda T. Honjo T. (2004). Production of superoxide anion and hydrogen peroxide by the red tide dinoflagellate Karenia mikimotoi. J. Biosci. Bioeng. 97, 212–215. 10.1016/S1389-1723(04)70193-0 16233617

[B59] Yuan J. Shiller A. M. (2001). The distribution of hydrogen peroxide in the southern and central Atlantic ocean. Deep Sea Res. Pt II 48, 2947–2970. 10.1016/S0967-0645(01)00026-1

[B60] Zafiriou O. C. (1990). Chemistry of superoxide ion-radical (O${}_{2}^{-}$) in seawater. I. pKa,sw^*^ (HOO) and uncatalyzed dismutation kinetics studied by pulse radiolysis. Mar. Chem. 30, 31–43.

[B61] Zhang Y. del Vecchio R. Blough N. V. (2012). Investigating the mechanism of hydrogen peroxide production by humic substances. Environ. Sci. Technol. 46, 11836–11843. 10.1021/es3029582 23046212

