# Supplementary material for: Species-Level Variability in Extracellular Production Rates of Reactive Oxygen Species by Diatoms
Source: Front Chem. 2016 Mar 30;4:5. doi: 10.3389/fchem.2016.00005 (PMC4812844; doi:10.3389/fchem.2016.00005)
Supplement: Supplementary file 1 [file DataSheet1.pdf]

Supplementary Material for
Species-level Variability in Extracellular Production Rates of Reactive Oxygen Species by Diatoms

Robin J. Schneider, Kelly L. Roe, Colleen M. Hansel, Bettina M. Voelker

S1. Details of the O_2^- measurements

S1.1 Experimental Set-up

The MCLA reagent was composed of 30 μM DTPA, 5 μM 2-methyl-6-[p-methoxyphenyl]-3,7-dihydroimidazo[1,2-a]pyrazin-3-one (MCLA) (TCI America), and 0.2 M MES hydrate buffer (Sigma), adjusted to pH 6.0 with ~0.074 M NaOH (Sigma). The flow injection system was set up to directly pump the ASW and MCLA reagent into a flow cell at a flow rate of 3 mL min^{-1} (Figure S1). The homemade flow cell was made of tygon tubing, which sat directly under a photomultiplier tube (model H9319-11, Hamamatsu). The PMT was set to integrate the signal over 20 ms, a collection time of 10 periods, with the default setting for PMT voltage, and two data points were collected every second. All measurements were made in a positive pressure clean hood.

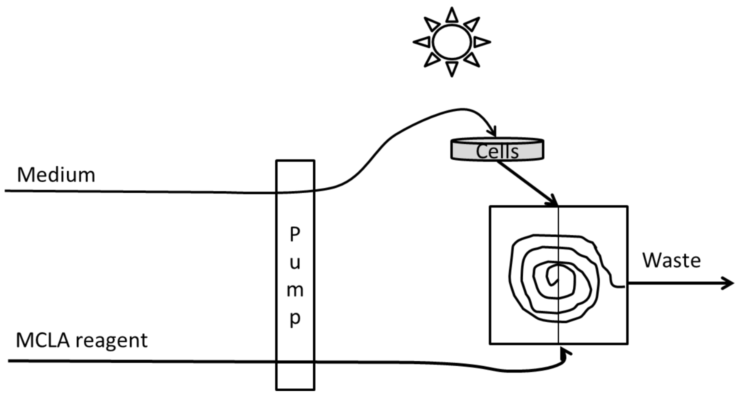

Figure S1. Schematic of O_2^- setup for the FIA system. O_2^- setup shows the experimental medium and MCLA reagent being pumped at 3.0 mL min^{-1} into the FIA flow cell where a photomultplier tube detects the chemiluminescence signal. The phytoplankton cells are immobilized on the filter where they could be exposed to light or dark conditions. The filter was positioned as close to the flow cell as possible with the filter disk parallel to the floor. All tubing except that used in the peristaltic pump was black PEEK tubing.

S1.2 Calibration

A primary O_2^- stock solution was made fresh for each calibration point by adding a small amount of KO_2 powder to a solution of 30 μM DTPA and 0.032 M NaOH (nominal pH 12.5) (Heller and Croot, 2010). The O_2^- concentration in the primary stock was calculated by measuring the absorbance of the solution before and after addition of 2.4 U mL^{-1} of Cu/Zn superoxide dismutase (Sigma) on a Black-comet UV/Vis spectrometer with a SL5 Deuterium/Halogen light source (Stellar Net Inc) at 240 nm and dividing the difference in absorbance by the molar extinction coefficient corrected for H_2O_2 absorbance at pH 12.5, 2183 cm^{-1} M^{-1} (Bielski *et al.*, 1985). A known volume of the primary O_2^- stock solution was withdrawn immediately after the absorbance measurement (before addition of superoxide dismutase) and transferred into a solution of 10 μM DTPA and 1 mM NaOH to make the working stock. A small volume of the working stock was then spiked into the ASW (at a ratio not exceeding 100 μL:

100 mL ASW), and the chemiluminescence signal was monitored over time. A maximum of 3 min passed between the absorbance measurement of the primary O_2^- stock and the addition of the working stock spike to the sample. Microsoft Excel's Solver function was used to minimize the sum of the squares of the differences between the actual data and the model to determine best fit values of $R_{BL,}$ $R_{t=0}$, and $k_{loss,O2-}$ in Equation (1) in the main text.

S.1.3 Experimental runs

The experimental run was started by pumping fresh ASW over an acid-washed 25-mm 0.45-μm cellulose acetate filter for ~4 min to get a steady baseline signal (region 1 in Figure S2), R_{ASW}. The pump was then briefly stopped (< 3 s) while the tubing was switched to the culture. The cells were loaded onto the filter through the pump, which took less than 2 min (region 2 in Figure S2). The pump was again briefly stopped (< 3 s) while the tubing was switched back to the ASW. The signal was then monitored for ~10 min (region 3 in Figure S2), with R_{cell} obtained as the average of the 500 counts taken at the end of this time period. A O_2^- spike was then added to the ASW from a freshly prepared working stock solution, as described for the calibration procedure above, and the signal was monitored for ~5 min (region 4 in Figure S2). Finally, superoxide dismutase (SOD) was added to the ASW at a final concentration of ~0.24 U mL^{-1} (~1.6 nM) (region 5 in Figure S2). R_{SOD} was obtained as the average of 100 counts taken as soon as the signal stabilized after the SOD addition.

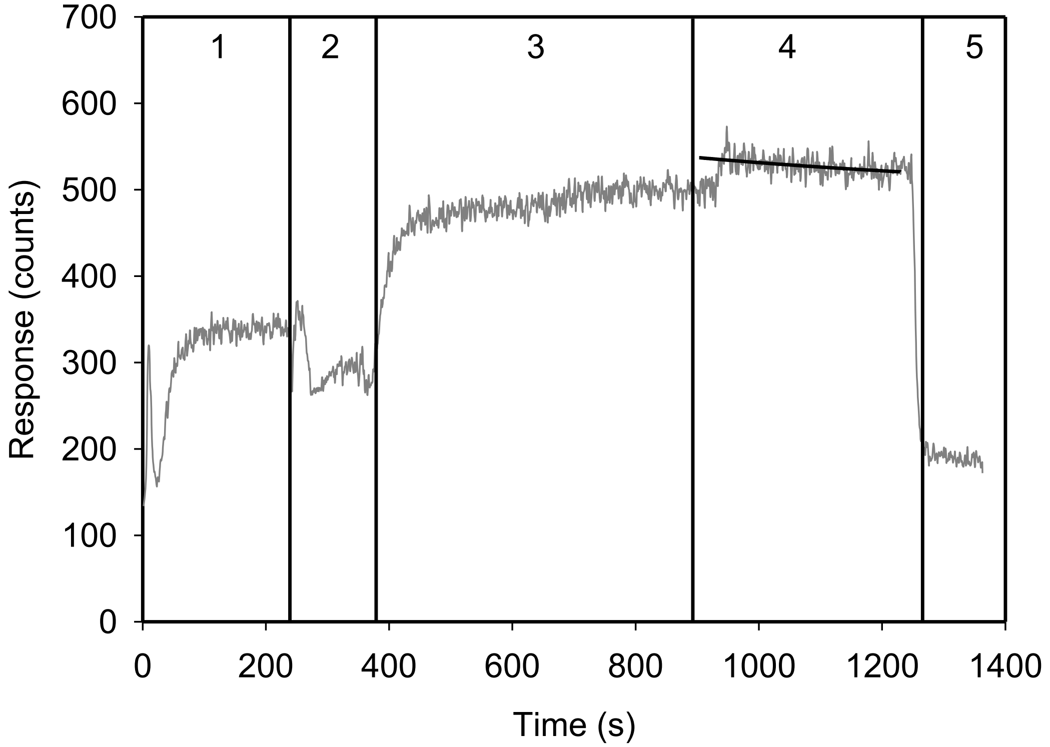

Figure S2. Example of a O_2^- run with phytoplankton cells. Region 1 shows signal when ASW is passed over the empty filter. Region 2 shows signal when cells are loaded onto the filter from F/2 medium. Region 3 shows the signal when ASW is passed over the cells. Region 4 shows the signal from the O_2^- spike (15% recovery) with the solid line showing the model fit to the spike. Region 5 shows the signal when ~0.24 U mL^{-1} (~1.6 nM) SOD is added.

S2. Details of the H_2O_2 measurements

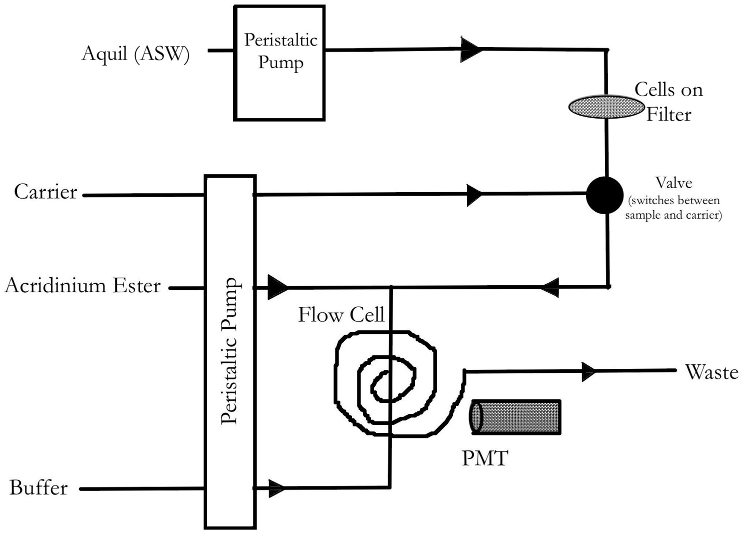

Figure S3. Schematic of flow-injection system for measuring H_2O_2. Setup shows the experimental medium being pumped at 0.6 mL min^{-1} by the first peristaltic pump, while the carrier, acridinium ester, and carbonate buffer are pumped by a second peristaltic pump at 2.0 mL min^{-1} into the flow cell where a photomultiplier tube detects the chemiluminescence signal.

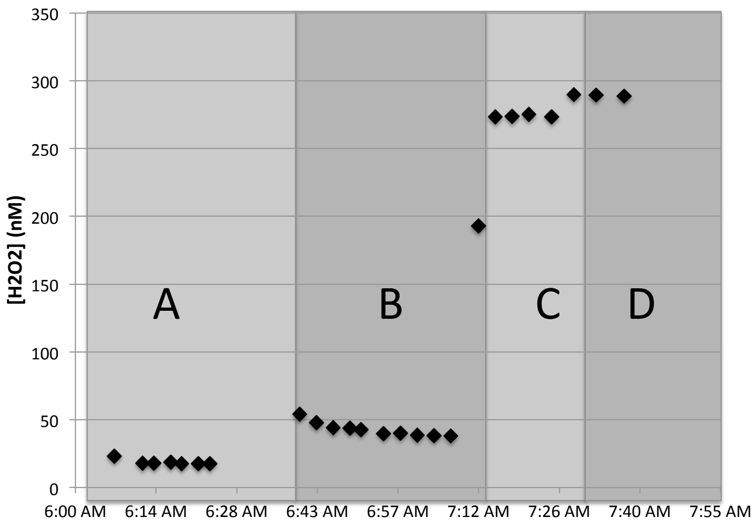

Figure S4. Example H_2O_2 run using data points from *T. pseudonana* on 7/7/2014, showing (A) unamended ASW passed over filter; (B) unamended ASW passed over cells; (C) ASW spiked with H_2O_2 passed over cells; (D) spiked ASW measured directly.

S3. H_2O_2 production rates for *C. cryptica* and *P. tricornutum*

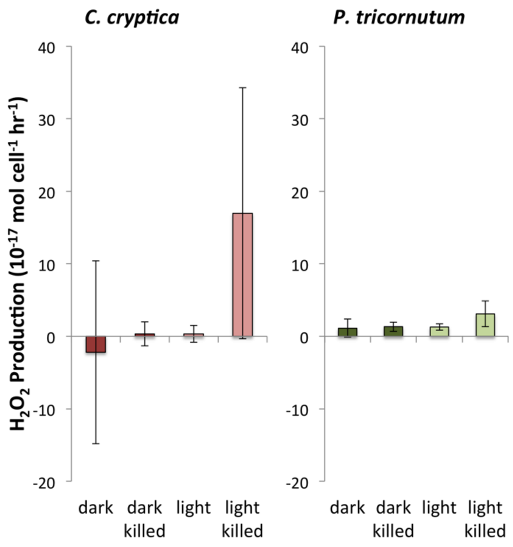

Figure S5. Calculated H_2O_2 production rates in *C. cryptica* and *P. tricornutum*. Error bars represent one standard deviation. None of the rates was significantly different from zero.

S4. Surface-area normalized production rates

To determine whether production rates of O_2^- and H_2O_2 were a function of cell surface area, surface-area normalized production rates were calculated as follows. Average cell surface area (SA_{avg}) for a given diatom was calculated by using average cell dimensions provided by the National Center for Marine Algae and assuming that diatoms were perfect cylinders (Table S1). Total cell surface area (SA_{tot}) in the filter was then calculated by multiplying average cell surface area by the estimated number of cells on the filter:

$$SA_{tot} = SA_{avg} d_{culture} V_{culture} \tag{S1}$$

where $d_{culture}$ is the density of the culture in cells mL^{-1} and $V_{culture}$ is the volume of culture loaded on the filter. Surface-area normalized production rates (Figures S6 and S7) were calculated by dividing observed O_2^- production, $[O_2^-]_{cell}$, and H_2O_2 production, $[H_2O_2]_{cell}$, by the total surface area, SA_{tot}.

Table S1. Cell size range data provided by the National Center for Marine Algae, and averages and surfaces used in the present study

Organism	Diameter (μm) Range	Diameter (μm) Average	Height (μm) Range	Height (μm) Average	Surface Area (μm^2)
T. weissflogii	10-12	11	12-22	17	1055
T. pseudonana	4-5	4.5	4-6	5	133
T. oceanica	4-12	8	6-10	8	603
P. tricornutum	3	3	13	13	137
C. cryptica	8-10	9	10-18	14	722

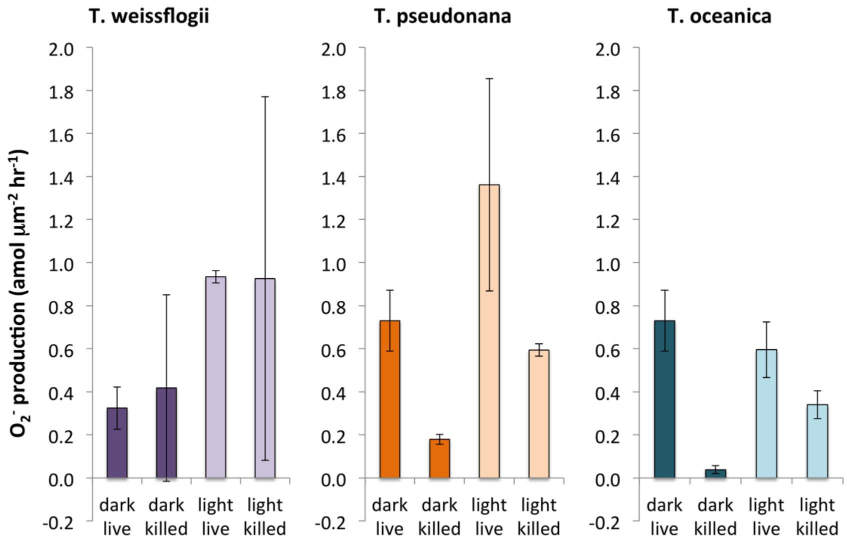

Figure S6. Calculated surface-area normalized O_2^- production rates in *Thalassiosira* spp. Error bars represent one standard deviation.

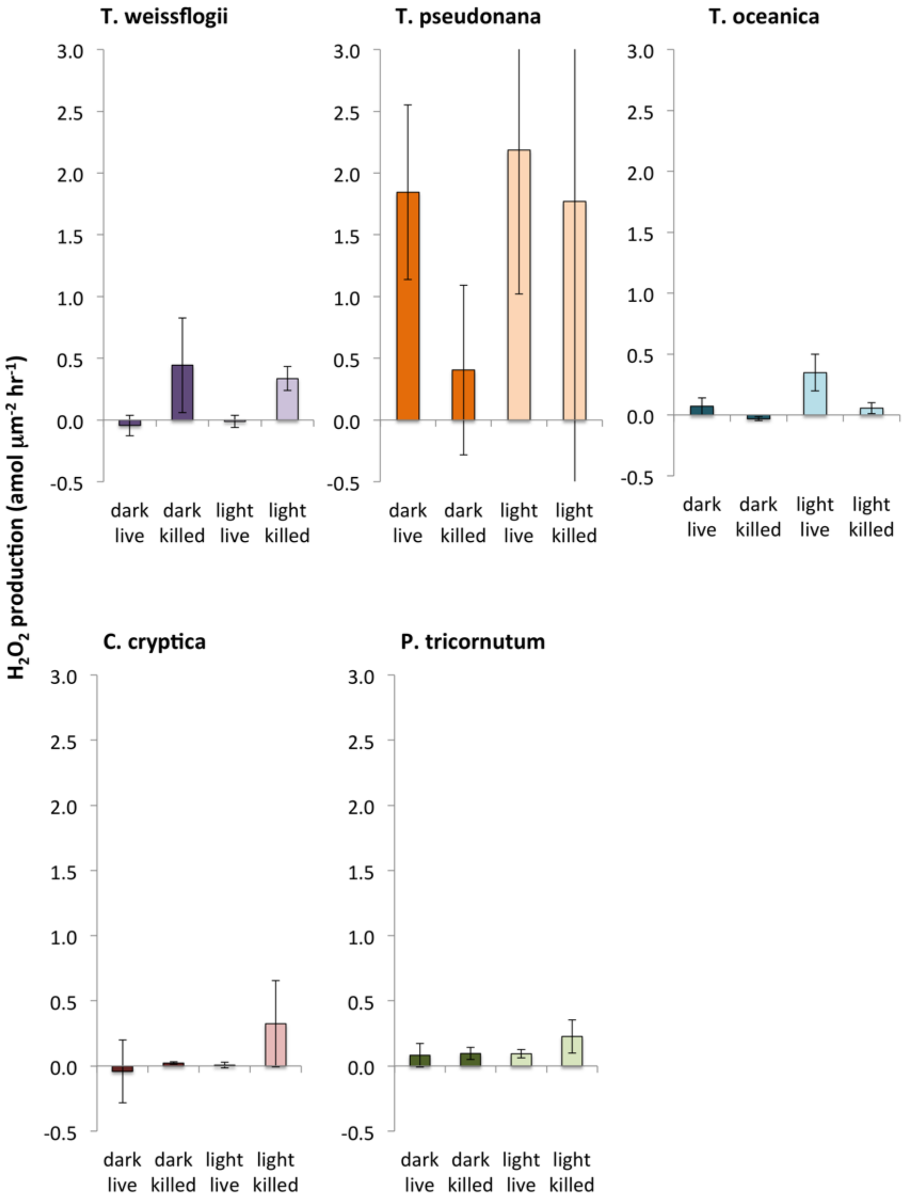

Figure S7. Calculated surface-area normalized H_2O_2 production rates in all five species of diatoms studied. Error bars represent one standard deviation. Error bars on *T. pseudonana* are cut off to maintain a similar scale to the other diatoms.

S5. Surface-area normalized ROS decay

To compare the relative abilities of different organisms to break down H_2O_2 and O_2^-, we converted our measured recovery percentages to decay coefficients. The equations for this conversion can be derived by treating the dead volume in the filter from the flow-through method as a well-mixed reactor at steady state (Hemond and Fechner-Levy, 2000).

Using the well-mixed reactor model, the change over time in the concentration of H_2O_2 within the filter, $[H_2O_2]$, can be written as:

$$\frac{d[\mathrm{H_2O_2}]}{dt} = m_{\mathrm{H2O2},in} - m_{\mathrm{H2O2},out} + \text{Production} - \text{decay} \qquad \text{(S2)}$$

where $m_{H2O2,in}$ and $m_{H2O2,out}$ represent the rate at which H_2O_2 is transported into and out of the filter, respectively, in units of nM hr^{-1}, while the production and decay terms represent the result of biological activity within the filter. Equation S2 can be rewritten as:

$$\frac{d[\mathrm{H_2O_2}]}{dt} = \frac{Q[\mathrm{H_2O_2}]_{in}}{V} - \frac{Q[\mathrm{H_2O_2}]}{V} + P'_{\mathrm{H2O2}} - k'_{loss,H2O2}[\mathrm{H_2O_2}] \qquad \text{(S3)}$$

where $[H_2O_2]_{in}$ is the concentration of H_2O_2 in the analytical medium entering the filter, Q is the flow rate in L hr^{-1}, V is the dead volume of the filter in L, P'_{H2O2} is the biological production rate in nM hr^{-1} and $k'_{loss,H2O2}$ is the first-order decay coefficient of the H_2O_2 within the filter. At steady state ($\frac{d[\mathrm{H_2O_2}]}{dt}$=0), Equation S3 can be rewritten as:

$$\left(\frac{\mathrm{Q}}{\mathrm{V}}\right)\{[\mathrm{H_2O_2}] - [\mathrm{H_2O_2}]_{in}\} = \mathrm{P}'_{H2O2} - \mathrm{k}'_{loss,H2O2}[\mathrm{H_2O_2}] \qquad \text{(S4)}$$

In the H_2O_2 experiments, $[H_2O_2]$ and $[H_2O_2]_{in}$ were measured as the quantities $[H_2O_2]_{unspiked,cells}$ and $[H_2O_2]_{unspiked,direct}$ in analytical medium without added H_2O_2, and as the quantities $[H_2O_2]_{spiked,cells}$ and $[H_2O_2]_{spiked,direct}$ in medium spiked with H_2O_2. Therefore, Equation S4 can be written for both spiked and unspiked conditions:

$$\left(\frac{Q}{V}\right)\left\{[H_2O_2]_{unspiked,cells} - [H_2O_2]_{unspiked,direct}\right\} = P'_{H2O2} - k'_{loss,H2O2}[H_2O_2]_{unspiked,cells} \quad \text{(S5)}$$

$$\left(\frac{Q}{V}\right)\left\{[H_2O_2]_{spiked,cells} - [H_2O_2]_{spiked,direct}\right\} = P'_{H2O2} - k'_{loss,H2O2}[H_2O_2]_{spiked,cells} \quad \text{(S6)}$$

Combining Equations S5 and S6 gives a solution for $k_{loss,H2O2}$:

$$k'_{\mathrm{loss,H2O2}} = \left(\frac{Q}{V}\right)\left\{\frac{[H_2O_2]_{\mathrm{spiked,direct}} - [H_2O_2]_{\mathrm{unspiked,direct}}}{[H_2O_2]_{\mathrm{spiked,cells}} - [H_2O_2]_{\mathrm{unspiked,cells}}} - 1\right\} \quad \text{(S7)}$$

which can be rewritten as

$$k'_{loss,H2O2} = \left(\frac{Q}{V}\right)\left\{\frac{1}{\mathrm{Rec}_{H2O2}} - 1\right\} \quad \text{(S8)}$$

where Rec_{H2O2} is the recovery calculated by Equation 7 in the main text.

Because of the rapid degradation of the O_2^- spikes in the analytical medium, the O_2^- equivalents to the steady-state quantities $[H_2O_2]_{spiked,cells}$ and $[H_2O_2]_{spiked,direct}$ could not be measured. We therefore assumed that our measured values of Rec_{O2-} were representative of steady-state conditions to calculate estimates of surface-area normalized decay coefficients for O_2^- using:

$$k'_{loss,O2-} = \left(\frac{Q}{V}\right)\left\{\frac{1}{\mathrm{Rec}_{O2-}} - 1\right\} \quad \text{(S9)}$$

The cell-surface area normalized decay coefficients for each ROS (Figures S8 and S9) were calculated by dividing k'_{loss} for each ROS by the total cell surface area in the filter as calculated by Equation S1. For cell-density normalized decay coefficients (Table S2), we divided k'_{loss} by the cell density on the filter instead.

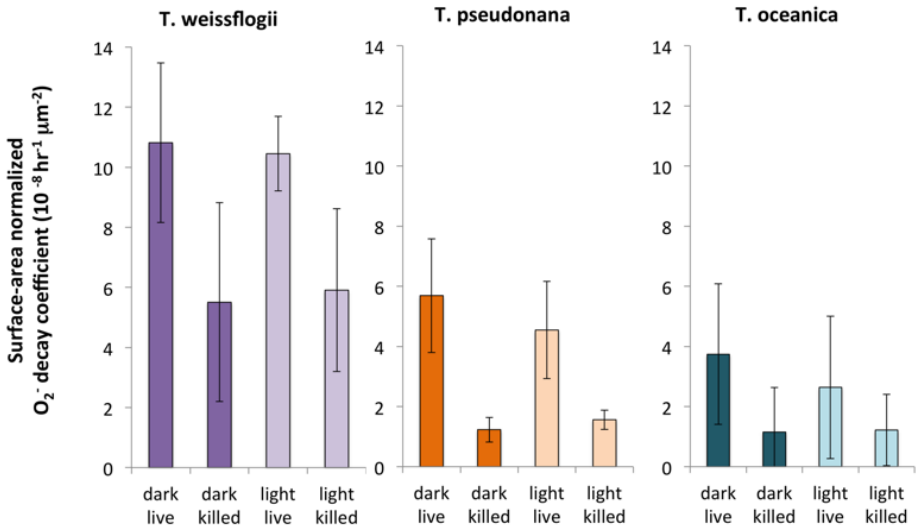

Figure S8. Calculated surface-area normalized O_2^- decay rate coefficients for *Thalassiosira* spp. Error bars represent one standard deviation.

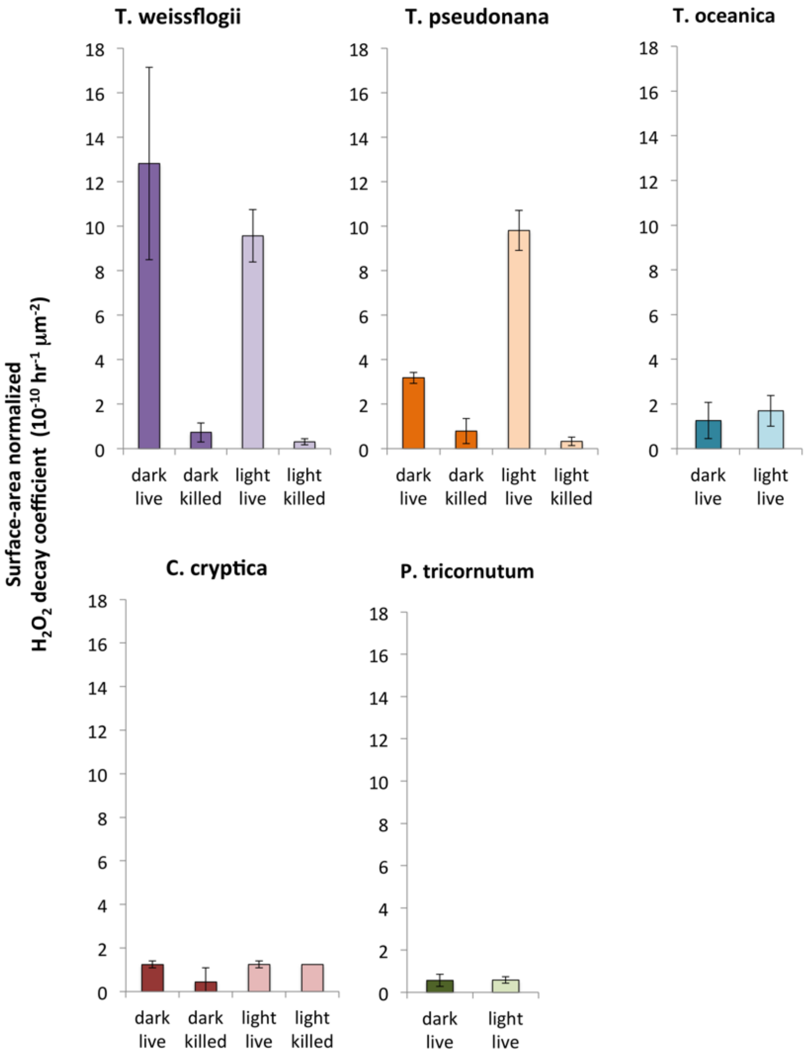

Figure S9. Calculated surface-area normalized H_2O_2 decay rate coefficients for all 5 diatoms studied. Error bars represent one standard deviation. Observed recoveries for killed controls of *T. oceanica* were over 100%, indicating insignificant decay.

Table S2. Cell-density-normalized decay rate coefficients for diatoms. Uncertainties represent one standard deviation.

	**O_2^- (x10^{-6} hr^{-1} (cell mL^{-1})$^{-1}$)**		**H_2O_2 (x10^{-8} hr^{-1} (cell mL^{-1})$^{-1}$)**	
	**Dark (live)**	**Light (live)**	**Dark (live)**	**Light (live)**
T. weissflogii	50.4 ± 12.4	48.7 ± 5.8	6.0 ± 2.0	4.5 ± 0.5
T. pseudonana	3.5 ± 1.2	0.3 ± 0.1	2.0 ± 0.2	6.0 ± 0.6
T. oceanica	6.8 ± 4.2	4.8 ± 4.3	2.3 ± 1.5	3.1 ± 1.2
C. cryptica	--	--	3.9 ± 0.5	3.9 ± 0.5
P. tricornutum	--	--	0.5 ± 0.2	0.5 ± 0.1

References

Bielski, B. H. J., Cabelli, D. E., Arudi, R. L., & Ross, A. B. (1985). Reactivity of HO2/O−2 Radicals in Aqueous Solution. *J. Phys. Chem. Ref. Data*, *14*(4), 1041–61. http://doi.org/10.1063/1.555739

Hemond, H.F., and Fechner-Levy, E.J. (200). Chemical Fate and Transport in the Environment, 2nd ed., Academic Press, San Diego, pp. 5-20.
